# Development of adaptive anoikis resistance promotes metastasis that can be overcome by CDK8/19 Mediator kinase inhibition

**DOI:** 10.1101/2023.12.04.569970

**Published:** 2023-12-06

**Authors:** Mehri Monavarian, Emily Faith Page, Resha Rajkarnikar, Asha Kumari, Liz Quintero Macias, Felipe Massicano, Nam Y Lee, Sarthak Sahoo, Nadine Hempel, Mohit Kumar Jolly, Lara Ianov, Elizabeth Worthey, Abhyudai Singh, Eugenia V Broude, Karthikeyan Mythreye

**Affiliations:** 1 Division of Molecular Cellular Pathology, Department of Pathology, O’Neal Comprehensive Cancer Center, University of Alabama, Heersink School of Medicine, Birmingham, AL, USA; 2 UAB Biological Data Science Core, The University of Alabama at Birmingham, Birmingham, Alabama, USA; 3 Department of Neurobiology, The University of Alabama at Birmingham, Birmingham, Alabama, USA; 4 Division of Pharmacology, Chemistry and Biochemistry, College of Medicine, University of Arizona, Tucson, AZ, 85721, USA; 5 Department of Bioengineering, Indian Institute of Science, Bangalore, 560012, India; 6 Department of Medicine, Division of Hematology Oncology, University of Pittsburgh School of Medicine Pittsburgh PA 15213; 7 Department of Electrical and Computer Engineering, University of Delaware, Newark, DE 19716, USA; 8 Department of Drug Discovery and Biomedical Sciences, College of Pharmacy, University of South Carolina, Columbia, SC 29208, USA

## Abstract

Anoikis resistance or evasion of cell death triggered by cell detachment into suspension is a hallmark of cancer that is concurrent with cell survival and metastasis. The effects of frequent matrix detachment encounters on the development of anoikis resistance in cancer remains poorly defined. Here we show using a panel of ovarian cancer models, that repeated exposure to suspension stress in vitro followed by attached recovery growth leads to the development of anoikis resistance paralleling in vivo development of anoikis resistance in ovarian cancer ascites. This resistance is concurrent with enhanced invasion, chemoresistance and the ability of anoikis adapted cells to metastasize to distant sites. Adapted anoikis resistant cells show a heightened dependency on oxidative phosphorylation and can also evade immune surveillance. We find that such acquired anoikis resistance is not genetic, as acquired resistance persists for a finite duration in the absence of suspension stress. Transcriptional reprogramming is however essential to this process, as acquisition of adaptive anoikis resistance in vitro and in vivo is exquisitely sensitive to inhibition of CDK8/19 Mediator kinase, a pleiotropic regulator of transcriptional reprogramming. Our data demonstrate that growth after recovery from repeated exposure to suspension stress is a direct contributor to metastasis and that inhibition of CDK8/19 Mediator kinase during such adaptation provides a therapeutic opportunity to prevent both local and distant metastasis in cancer.

## INTRODUCTION

Metastasis is the cause of the majority of cancer related deaths across cancer types and subtypes and remains largely incurable. An appreciated cause of metastasis is the acquisition and development of phenotypic characteristics that enable the survival of tumor cells after detachment from the extracellular matrix and primary tumor for local dissemination, and in circulation to distant sites. ^1–3^ Despite extensive characterization and defining essential features of metastatic cells, preventing the development of metastatic features remains an underdeveloped therapeutic opportunity.

Ovarian cancer is the most devastating of gynecological cancers (fifth in cancer deaths among women) and an archetypal example of a cancer that leverages the metastatic hallmark of anoikis resistance for both trancoelomic/intraperitoneal and distant metastasis. ^4–8^ Accumulation of malignant ascites in the peritoneal cavity occurs as disease progresses, and this process necessitates suspension survival and/or bypass of cell death mechanisms in tumor cells. ^9–12^ The precise mechanisms by which cells acquire such anoikis resistance and adapt for apoptosis avoidance as a result of detachment stress remains a subject of intense investigation. Suspension culture studies have been used extensively to understand such mechanisms, however most of them focus on single time points or long term suspension culture studies, in several instances focusing on the enrichment of cancer stem cells in specialized media. Despite potential limitations of such studies, tumor intrinsic signals and pathways, that change the ability of cells to undergo cell death upon matrix detachment (anoikis) ^13–17^ have been identified. These include but are not limited to either transcriptional upregulation of critical survival genes, transient expression changes in genes associated with antioxidant defense, and repression of genes that may inhibit survival to directly promote or repress **i**ntra **p**eritoneal (ip) OC tumor growth as described in many studies. ^18–22^ Coordinated regulation of specific reprogramming processes such as epithelial to mesenchymal transition (EMT) ^23,24^, cadherin and integrin switching ^25^ by oncogenic pathways such as Ras/Erk, PI3k/AKT, Rho, MYC and TGF-β pathways also impact survival and growth under anchorage independence. ^26^ However only few studies^27 28^directly compare anoikis sensitive and resistant models, which is required to understand how resistance can be reached, or specifically prevented.

In accordance with the significance of transcriptional changes to overall cancer progression, selective and potent inhibitors of RNA polymerase II transcription asssociated kinases ^29^ have emerged and are currently being evaluatd in the clinic. CDK8 and CDK19 Mediator kinases are non cell cycle regulating kinases that both positively and negatively regulate transcription ^30–32^ and inhibition of their kinase activity can change reprogramming events including EMT ^24^, cell fate ^33^ and gene expression responsive to various signals and stressors. ^30,32^ Notably, CDK8/19 inhibitors have reached clinical trials for solid tumors and leukemias (clinicaltrials.gov NCT03065010, NCT04021368, NCT05052255, NCT05300438), however no prior study has assessed their utility in ovarian cancer models.

In this study we describe a model system that utilizes repeated exposures to detachment stress followed by attached re-growth in order to mimic potential in vivo scenarios, and delineate the impact of such repeat exposures to suspension stress on the development of anoikis resistance in ovarian cancer models. Our detailed phenotypic and transcriptomic characterisation of the anoikis adapted cells reveals non-genetic, transcriptional reprogramming, concomitant with a more aggressive phenotype in vitro and in vivo. Using such models we define a novel therapeutic strategy for prevention of anoikis resistance associated metastasis by specific targeting of transcriptional reprgramming using inhibitors of CDK8/19 Mediator kinase.

## RESULTS

### Attachment-detachment cycles confer anoikis sensitive ovarian cancer cells with resistance to cell death in suspension.

**Figure 1.**
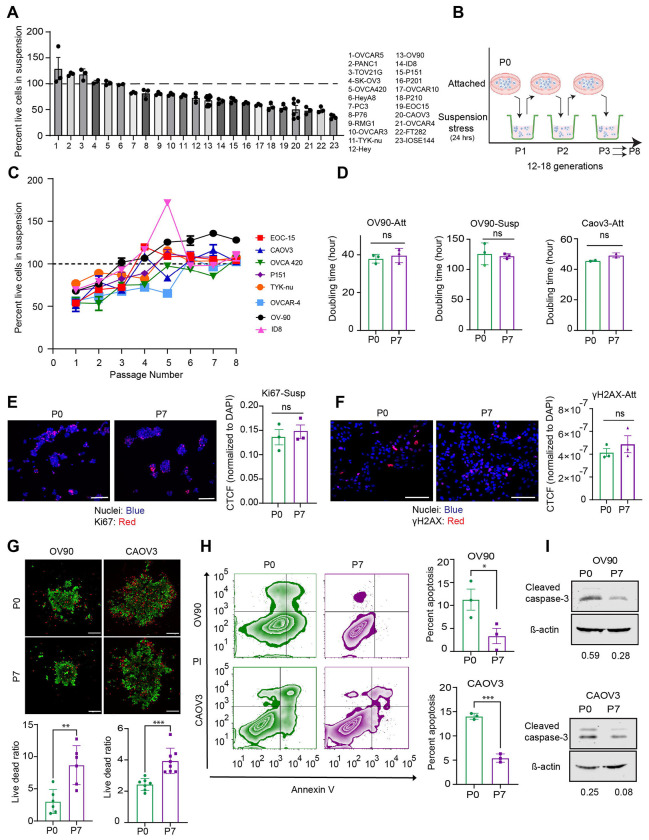
Development of anoikis resistance upon cyclic exposure to suspension stress (A) Percent live cells relative to initial plating number, of indicated cell models after 24 hours in suspension assessed by Trypan blue exclusion assay. (n>3 for all cell lines). Mean ± SEM (B) Schematic of cycles of attached growth followed by suspension conditions for testing development of resistance to anoikis. (C) Percent live cells in suspension of indicated anoikis sensitive (AnS) cells measured by trypan blue staining after 24 hours in suspension following cyclic gain and loss of attachment as in (B). (n= 3-12 biological replicates per cell line). (D) Doubling time of indicated cells either in attached growth conditions or in suspension culture calculated over a 7 day or 10-day period respectively using an SRB assay. (Parental: standard culture conditions or AnR: anoikis resistant cells from between passages P7-P9 from C) (n=2 biological replicates for CAOV3 and n=3 for OV90). Mean ± SEM, ns p > 0.05. (E) Representative confocal images (left) and quantitation (right) of the corrected total cell fluorescence (CTCF) of Ki67 (red) normalized to CTCF of DAPI of OV90 AnS and AnR derivative cells cytospun after 24 hours in suspension. ns p > 0.05, unpaired t-test, Mean ± SEM. Scale bar: 100 μm. (n= 3 biological trials, each trial includes quantitation of four 20X fields) (F) Representative widefield immunofluorescence images (left) and quantitation (right) of CTCF of γH2AX (red) normalized to CTCF of DAPI from OV90 parental and AnR derivative cells in standard attachment culture conditions. ns p > 0.05, One-way ANOVA followed by Tukey’s multiple comparison. Scale bar: 100 μm. (n= 3 biological trials, each trial includes quantitation of a minimum of six 20X fields). (G) Representative live dead confocal images (upper panel) of indicated AnS and AnR derivative cells cultured for 24 hours in ultra-low attachment plates. Scale bar: 200 μm. Live dead cell ratio in the spheroids assessed by Calcein AM (green, live cells) and ethidium homodimer dye (red, dead cells). (n= 6). Mean ± SEM, ** p < 0.01, One-way ANOVA followed by Tukey’s multiple comparison. (H) Flow cytometry analysis and quantitation (right graph) of percent apoptosis determined using annexin V and PI staining in indicated AnS and AnR derivative cells after 24 hrs in ultra-low attachment plates. (n= 3 biological replicates). Data are Mean ± SEM, *** p < 0.001, One-way ANOVA followed by Tukey’s multiple comparison. (I) Representative western blot for cleaved caspase 3 in indicated AnS and AnR derivative cells after 24 hrs in ultra-low attachment plates. Quantitation of cleaved caspase 3 normalized to β-actin shown below. (n= 2).

To study how cells develop the ability to evade cell death upon loss of attachment also referred to as anoikis resistance, we first broadly screened a panel of established cancer cell lines that span a spectrum of commonly used ovarian cancer cell line models, a pancreatic cancer cell line (PANC1), prostate cancer cell line (PC3), a primary population (non immortalized) of tumor cells derived from the ascitic fluid of an ovarian cancer patient (EOC15) and three previously established non oncogenic fallopian tube epithelial cell lines (FT282 and P201 and P210). ^34,21^ The goal was to systematically define the anoikis spectrum, and identify cell lines that exhibited sensitivity to loss of attachment as measured by the percentage of live cells after plating in poly Hema coated ultra-low attachment conditions in their respective growth media for a fixed time of 24 hrs. All cell lines were plated at the same cell density in their growth media to minimize stress from other variables. Cell lines exhibited a range of live cells in suspension relative to the initial plating numbers (36.1 % for IOSE144 to 125.2% for OVCAR5, Fig 1A, hereby referred to cell viability). Models exhibiting less than 100% live cells plated into suspension at 24 hrs were designated as ‘anoikis sensitive’ and models exhibiting 100% or more live cells plated into suspension at 24 hrs as ‘intrinsically anoikis resistant’. A subset of cell lines that exhibited resistance at 24 hrs, were further assessed for changes in viability for upto 72 hrs in suspension, to test if changing the time in suspension would alter their overall ability to survive in suspension. HEYA8, PANC1 and TOV21G cells retained their live cell percentages and anoikis resistance for upto 72 hrs. However OVCA420 cells increased their cell death and thereby less live cells over time with increase in cell death seen at 72 hrs in suspension (Supp Fig 1B). Hence, for OVCA420 alone, 72 hrs was used as an anoikis sensitivity time point in all subsequent experiments. EOC15 represents a primary population of non-immortalized epithelial cells obtained from patient ascites ^20^ that had been maintained in attachment culture (Supp Fig lA) and also exhibited measurable cell death in suspension at 24 hrs (Fig 1A).

Since the intrinsically resistant cell lines were resistant a priori, we asked whether sensitive cell lines were capable of developing resistance or were permanently and irreversibly sensitive to loss of attachment. To test this, we aimed to simulate a potential in vivo scenario of intraperitoneal and distant metastasis where cells are likely subjected to detachment stress followed by attached growth ^35^, and hence we exposed the cells to cyclic attachment detachment conditions where cells were exposed to suspension cultures for 24 hrs and expanded in attached cultures (Fig 1B). Of the 9 cell lines (P0) that exhibited <100% viability and identified as anoikis sensitive (AnS) at the first exposure to suspension cultures (P1), two types of responses to the cycles of attachment and detachment (Fig 1B) were observed. (i) 2/10 cell lines tested, acquired resistance (≥100% live cells) to loss of attachment after 3-4 attachment – detachment cycles (Supp Fig 1C), however were unable to maintain this resistance with the population folding back to sensitivity through subsequent cycles of attachment (2D) and suspension conditions (HEY, OVCAR3, Supp Fig 1C). (ii) on the other hand, 7/10 cell lines tested were able to significantly overcome cell death in suspension and most notably maintain such a resistance to cell death in suspension for additional rounds of attachment and detachment cycles (Fig 1C). While the 7 cell lines in Fig 1C differed to some extent in the number of attachment detachment cycles they took to reach and maintain near 100% or higher live cell viability in suspension, the number of cycles and timeline to stable anoikis resistance (AnR) was reproducible for a given cell line, across multiple trials. The generation time for the population to reach 100% viability in suspension and maintain such a viability was calculated for two cell lines OV90 and CAOV3 and was found to be 10.4 generations for OV90 and 10.5 for CAOV3 (P4 for OV90 and P6 for CAOV3 in Fig 1C).

We assessed if the increase in viability was due to changes in proliferation rate and/or population doubling times, or DNA damage that could lead to senescence over time that could affect cell viability. Proliferative differences between the resistant, (anoikis resistant: AnR, P6 or higher) or parental/sensitive, (anoikis sensitive: AnS, P0 or after exposure to one round of suspension- P1) cells were determined under both standard attached conditions and suspension conditions (assessed over a 10 day period in regular attached growth plates or in ultra-low attachment plates). No significant differences in doubling time for the parental (P0) and isogenic AnR (P6-8) cells in both 2D attached and suspension conditions were noted (Fig 1D). CAOV3 could not be assessed in suspension for 10 days due to extensive cell death. The proliferative index of the AnS (P0) and AnR cells in suspension as measured by Ki67 (Fig 1E) and DNA damage as a precursor to senescence at steady state attached conditions as determined *γ*H2AX staining (Fig 1F) indicated no significant differences between the AnS and AnR cells. In contrast, live/dead staining of both OV90 and CAOV3 parental and AnR cells in suspension (Fig 1G), and annexin V and PI staining followed by flow cytometry (Fig 1H) indicated a significant increase in the live/dead ratio (2.8 times higher for OV90 and 1.6 times higher for CAOV3) and reduced apoptosis (3.36 times lower in OV90 and 2.59 times lower in CAOV3) in AnR cells as compared to the parental AnS cells. Western blot analysis of cleaved caspase 3 from cells collected after 24 hrs in suspension also confirmed reduced cell death in the isogenic AnR cells as compared to parental P0 cells (Fig 1I).

To further evaluate if development of anoikis resistance as a result of cyclic exposure to loss of attachment following by attached growth (Fig 1B), mimicked the development of anoikis resistance in vivo in both immunocompromised and immunocompetent models, we injected OV90 cells and mouse ID8 cells ip into immunocompromised and immunocompetent mice respectively. Ascites fluid at the end point was used to collect cells that were then expanded in 2D for one passage followed by measurement of anchorage independent survival in suspension after 24 hrs. We find that both OV90 and ID8 cells from mouse ascites were resistant to cell death in suspension to similar extents as in vitro adapted AnR OV90 and AnR ID8 cells (Supp Fig 1D). These data suggest that AnR cells adapted to anoikis in vitro show a survival pattern similar to in vivo adapted AnR cells from ascites. Moreover, these cells don’t exhibit significantly higher or lower doubling times in vitro. Instead, they display resistance to the stress of detachment and reattachment cycles

### Acquired resistance to cell death in suspension is adaptive and reversible.

**Figure 2.**
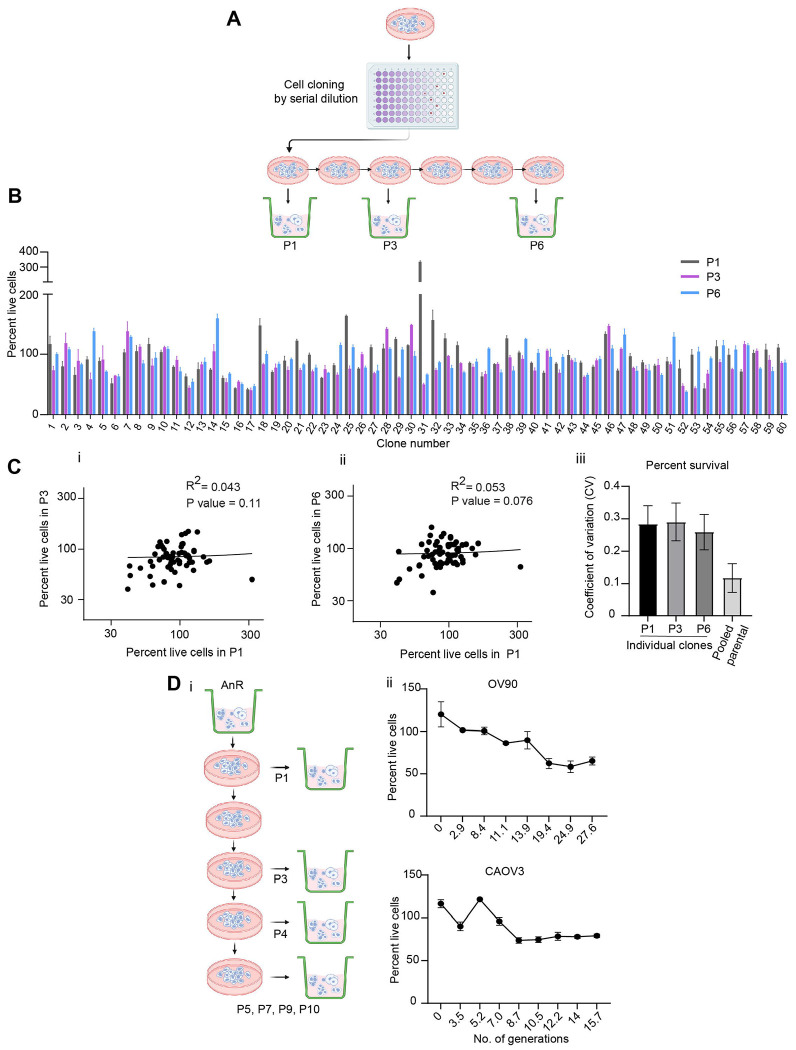
Acquired anoikis resistance represents a transient memory state in the population. (A) Scheme of single clone expansion from OV90 cells, followed by assessment of survival of the individual clones upon suspension culture in ultra-low attachment plates for 24 hrs in regular growth media at the indicated passages. (B) Percent survival of individual clones assessed in according to the scheme in (A). (n= 60 clones assessed). (C) (i) Linear regression correlation analysis of the survival of the individual clones in suspension P1 versus P3 or (ii) in P1 versus P6. (iii) Coefficient of variation in the survival in suspension, calculated for the individual clones at indicated passages along with the biological variation in the parental OV90 population which contains the pooled clones. Bootstrapping was used to obtain a 95% confidence interval. (D) (i) Experimental set up to determine the stability of the adapted AnR cells maintained in attached conditions and tested for survival in suspension at the indicated passages after attached expansion. (ii) Percent survival in suspension tested as in (i) plotted against the number of generations expanded in attached growth for OV90 and CAOV3 cells.

To determine whether such acquired resistance in the population was due to clonal selection, genetic/mutation driven, or non-genetic and transient in nature, we used a modified Luria-Delbrück fluctuation analysis. Several recent studies have modified and applied this classical Luria-Delbruck fluctuation test to probe cancer drug resistance mechanisms wherein single-cell derived clones were exposed to targeted drug therapy, and clone-to-clone fluctuations in the number of surviving cells were used to show that individual cells could reversibly switch between drug-sensitive and drug-tolerant states even before therapy. ^36–41^ We explored if a similar model of transient switching between cellular states could drive anoikis resistance. First, we determined the fluctuations in survival in suspension across single clones in the parental OV90 population (Fig 2A n=60 single clones). Individual clones were expanded for 20 generations prior to determining live cell viability in suspension upon detachment stress induced by plating cells in polyhema coated ULA plates (Fig 2A, B; P1 survival, grey bars). Clones were maintained in 2D (attached) cultures and percentage of cell viability of the expanded clonal population was quantified after three additional passages in 2D (P3) and again after a total of 6 passages in attached growth (Fig 2A, B, P3 and P6 survival). If the clones switched between states of sensitivity (<100% survival in suspension) and resistance (≥ 100%), then these data would suggest that the resistant state was non-heritable. We find no statistically significant correlation in the survival over time for the individual clones (Fig 2C i.ii) which had doubling times ranging from 39 hrs for the fastest to 62 hrs for the slowest (based on doubling time for 10 random clones assesed over 10 days, Supp Fig 2A) suggesting the absence of fixed clonal states with respect to survival in suspension. Our data also show that the statistics of the fraction of surviving cells in suspension across clones remains consistent for different passages with a mean survival fraction of ≈ *0.9*, and interclonal fluctuations as quantified by the coefficient of variation (CV; defined as the standard deviation divided by the mean) to be in the range of *0.25 – 0.3.* (Fig 2C.iii). These observed fluctuations were found to be higher than the fluctuations in the survival of the parental population in suspension which represents the pooled clones (Fig 2C.iii) as measured by quantifying the CV of cell survival between biological repeats (n=11 of OV90 cells from bulk culture placed in suspension). If every single cell responds purely randomly to stress then we would expect the fluctuations across clones to be similar to the noise in the population. However the difference in the noise as determined from the CV data suggest a form of memory in the clones, or the presence of pre-stress cellular states during clonal expansion that lead to single cell differences in their detachment stress responses driving anoikis resistance

To experimentally test memory in the population, we assessed the stability of the acquired resistant state in the overall population in two cell lines (OV90 and CAOV3) by maintaining and propagating the acquired isogenic resistant cells (AnR cells) in standard attached growth conditions for several generations (no stress). We then assessed cell death in suspension at various time points by rechallenging the resistant cells to suspension stress for 24 hrs (Fig 2D.i). We find that maintaining cells in attached conditions for between 11-14 generations for OV90 and 8-9 generations for CAOV3, led to cells regaining their sensitivity to loss of attachment (AnS) reaching the near original sensitivity levels of the parental population (Fig 2D.ii). While individual cell lines differed in the time required to regain sensitivity, this interval was reproducible across replicates (Fig 2D.ii).

We thus used previously developed analytical formulas ^38,40,41^ to predict what levels of fluctuations would be expected from switching between an anoikis-sensitive state (cell death in suspension) and an anoikis-resistant state (cell survival and proliferation in suspension). If *f* is the fraction of cells in the resistant state then

$$fe^{\gamma T}=0.9$$

where *T = 24 hr* and growth rate $\gamma =\frac{Ln\;2}{T_{d}}hr^{-1}$ where *T_d_* is the cell doubling time of anoikis resistant cells in suspension that is experimentally determined. Thus, given a value of *T_d_*, the fraction of resistant cells can be computed from the above equation. We used the cell-doubling time of the OV90 population in suspension as *100 hrs*, (Fig 1D) which results in *f ≈ 0.76*. Using equations derived previously, ^41^ individual clonal expansion in attached culture for 20 generations before the first survival test in suspension (Fig 2A,B **P1)**, and a transient heritability of approximately 10-11 generations for the resistant state (Fig. 2D), we obtain the model predicted fluctuation values to be much less than of 0.01 and 30-fold less than the observed fluctuations of *0.25 – 0.3* for all biologically-relevant values of *T_d_* greater than equal to *38 hrs* (OV90 cell doubling time in 2D) (Fig 1D). Thus, reversible switching between these two phenotypic states cannot explain the observed clone-to-clone variations in surviving cells. Considering a doubling time of *100 hrs* in suspension, a variation of 145% in the effective growth rate of single cells (i.e., a CV of 1.45 in *γ*) is needed to capture the observed inter-clonal fluctuations in the fraction of surviving cells. These data are hence not concordant with a classic two state model as proposed for chemoresistance ^42^and indicate the presence of likely more than two states in the population with respect to anoikis resistance, but are however consistent with non-genetic effects on adaptation of cells as they develop resistance to the biological stress of loss of attachment (anoikis resistance). The non genetic adaptation was also confirmed by whole exome sequencing. The total number of genes that were mutated in any given replicate was found to be 2860. However, no significant differentially mutated genes (using a p value of p < 0.05 and Fisher’s exact test) were found between the two populations of cells (Top 50 mutated genes based on total number of mutations present in OV90 cells is shown in Supp Fig 2B.

### Adapted anoikis tolerant ovarian cancer cells are more chemoresistant and metastatic in vivo.

**Figure 3.**
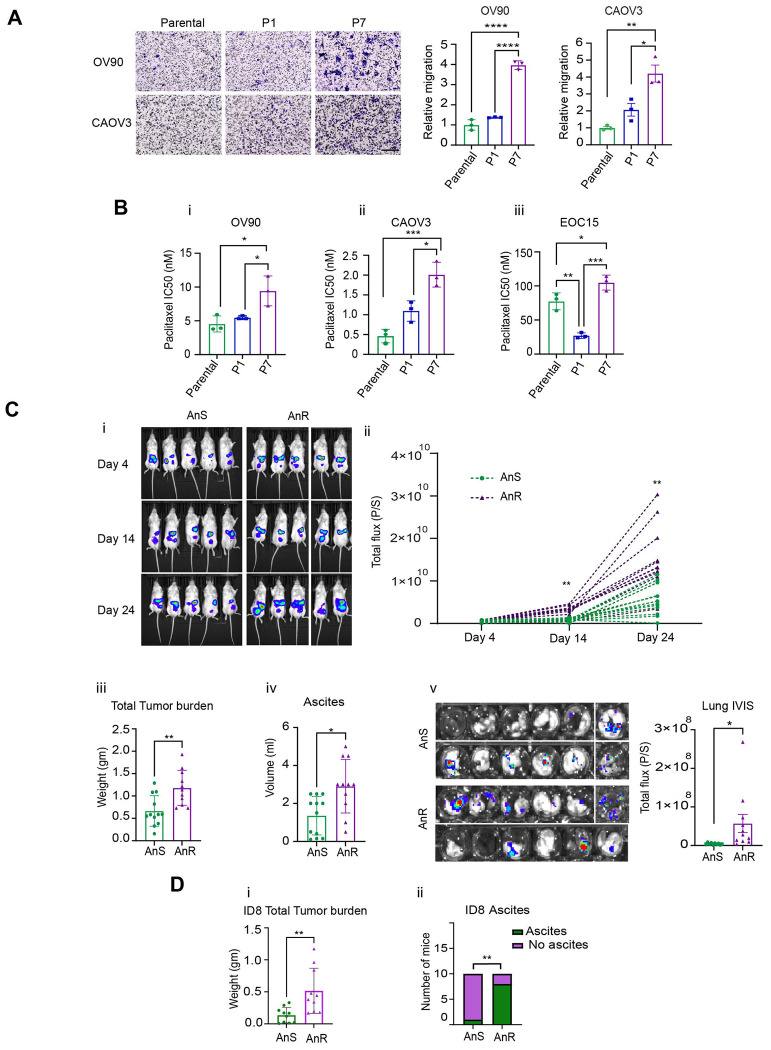
Acquired anoikis resistance leads to increased invitro migration, chemoresistance and in vivo intraperitoneal growth in human and mouse models (A). Representative images (left) and quantitation (right graph) of indicated parental, P1 (cells expanded after one exposure to suspension culture) and P7 (anoikis resistant/AnR) cells on fibronectin coated transwell filters after migration for 24 hrs. (n= 3). Data are mean ± SEM. ns p > 0.05, * p < 0.05, ** p < 0.01, **** p <0.0001. One-way ANOVA followed by Tukey’s multiple comparison. (B). IC50 to paclitaxel of (i) OV90, (ii) CAOV3 and (iii) immortalized EOC15 cells determined under steady state attached conditions of indicated cells after 72 hrs using an SRB assay. Data are mean ± SEM. ns p > 0.05, * p < 0.05, ** p < 0.01, **** p <0.0001. One-way ANOVA followed by Tukey’s multiple comparison. (C). Whole body luminescence (BLI) (i) representative images and (ii) quantification of total flux over time of NOD-SCID mice injected with 5 million live OV90 parental (P0/AnS) or anoikis resistant (P7/AnR) cells. Data are mean ± SEM. ns p > 0.05, ** p < 0.01, *** p <0.001, Two-way ANOVA followed by Tukey’s multiple comparison (iii) Total tumor weight (gms) and (iv) total ascites volume ml) from mice receiving P0 (AnS) or P7 (AnR) OV90 cells as indicated, analyzed between days 39-40. (v) Luminescence images of explanted whole lungs (left) and quantitation of total luminescence flux of the lungs from mice receiving either P0 or P7 OV90 cells at days 39-40. n=12 for AnS/P0 and 11 for AnR/P7. All data are Mean ± SEM; * p < 0.05, ** p < 0.01, unpaired t test. (D). Analysis of (i) total tumor weight or (ii) volume of retrievable ascites from C57BL6 mice, ip injected with either parental AnS (P0) murine ID8 or AnR (P8) ID8 cells between day80-82. n=10. Data are Mean ± SEM, ** p < 0.01, unpaired t test.

Prior studies have implicated anoikis resistance as a phenotype of metastatic and chemo resistant cells. ^43,5,44 45^ Given that reversion to the original anoikis state occurred in the absence of stress (Fig 2D) we tested if such an acquired ‘tolerance’ rather than permanent resistance (intrinsic), was sufficient to alter in vitro tumorigenic properties. A transwell migration assay revealed that OV90-AnR cells (cells derived from P7) were able to undergo significantly higher migration through fibronectin (Fig 3A) as compared to the parental population (P0), and also as compared to cells expanded after exposure to a single round of suspension culture (P1, Fig 3A). Increased migration of AnR cells was seen not just in OV90 cells, but also in CAOV3 cells (Fig 3A). The increased migration in P7 as compared to P1 also indicate that one round of an attachment detachment cycle is not sufficient to stimulate significant cell migration and was seen across additional isogenic pairs tested including EOC15, p151 and OVCA420 (Supp Fig 3A). All tested pairs revealed increased migration for the AnR isogenic derivatives only, demonstrating increased motility as a feature of acquired anoikis resistance. Assessing the chemosensitivity of the cells to standard of care chemotherapeutics including cisplatin, paclitaxel, and doxorubicin revealed that AnR cells across cell line models had significantly higher IC50 concentrations for paclitaxel (Fig 3B i–iii). Increased IC50 for paclitaxel was also seen for the AnR cells in suspension cultures (Supp Fig 3B). However no significant differences were observed for doxorubicin and cisplatin **(**Supp Fig 3C).

In order to assess if acquired anoikis resistance was sufficient to increase intraperitoneal (ip) growth and metastasis of tumor cells, we injected live OV90 parental luciferase expressing cells (parental/P0), or cells expanded under attached conditions after development of anoikis resistance (AnR/P7), into the peritoneal cavity of NOD-SCID mice. Whole body bioluminescence imaging (BLI), revealed a significant increase in overall ip. tumor burden over time in mice receiving AnR cells as compared to mice receiving parental (P0) cells (Fig 3C i,ii). BLI was monitored until day 37 near the end point of the studies, however ascites accumulation which began at ~day 25, precluded reliable BLI, and hence BLI data until day 24 are presented (Fig 3C i,ii). The study was terminated between day 39-40 when mice from the P7/AnR group were found to be moribund. End point total tumor weight in the peritoneal cavity of mice receiving P7 expanded cells (AnR) was twice as much as compared to in the mice receiving the parental population (AnS) (Fig 3C iii) concomitant with higher volume of ascites (Fig 3C iv) and burden in the mesentery (Supp Fig 3D i). Strikingly, animals receiving AnR cells (P7) had higher extent of lesions in the lung as determined by BLI imaging of explanted lung tissues as compared to the parental cells (P0) (Fig 3C v).

Since the in vitro derived AnR cells mirror suspension survival of in vivo derived AnR cells from human (OV90) and mouse origin (ID8), (Supp Fig 1D), we tested if acquired anoikis resistance was sufficient to increase intraperitoneal (ip) growth and metastasis of tumor cells in the presence of an immune system as well. We injected live ID8 parental cells (parental/P0), or cells expanded after development of anoikis resistance (AnR/P7) in vitro, into the peritoneal cavity of C57BL6 mice. The study was terminated when mice from the P7/AnR ID8 group exhibited signs of being moribund. Parental ID8 cells formed floating tumors as previously described ^14 46^ (Fig 3D). Adapted ID8 AnR cells produed significantly higher disease burden as evident from the higher overall measurable tumor burden (Fig 3Di) as seen in the mesentric regions (Supp Fig3D ii**),** omentum (Supp Fig 3D iii) and the number of mice with measurable ascites (Fig 3D ii). Lung metastasis was however not evident from pathological assessments. These data together demonstrate that repeated cycles of exposure to suspension stress followed by attached growth leads to adaptation that is sufficient to significantly promote aggressive disease in vitro and in vivo mimicking disease spread in stage IV ovarian cancer patients.

### Development of adaptive anoikis resistance is concomitant with transcriptional reprogramming over time.

**Figure 4.**
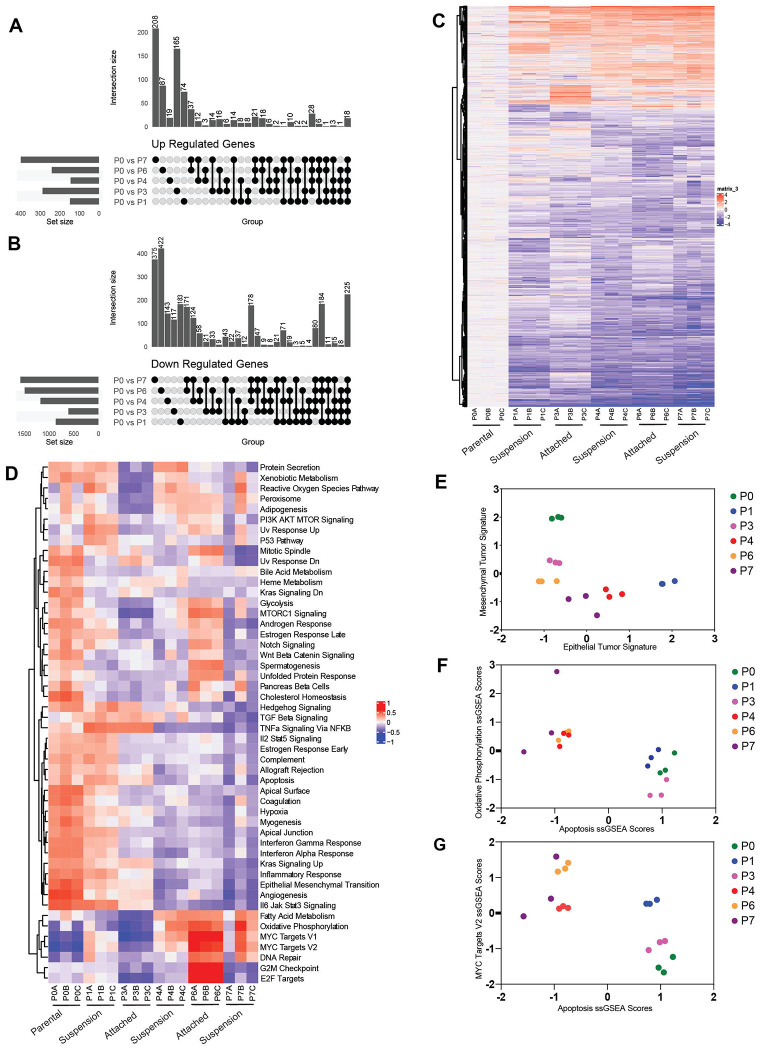
Trancriptional changes and pathway alterations during adaptation to anoikis over time. (A). UpSet plots highlighting the number of Up Regulated Genes (A) (p value < 0.05 and L2FC >1.5) and Down Regulated genes (B) (p value < 0.05 and L2FC < −1.5) in OV90 across the time point comparisons P0 vs P1, P0 vs P3, P0 vs P4, P0 vs P6, and P0 vs P7 as indicated. (C) Heatmap for the individual samples using Log2FC as calculated for individual biological replicates for parental (P0 cells) (P0A-C), P1 (P1A-C), P3 (P3A-C), P4 (P4A-C), P6 (P6A-C), and P7 (P7A-C). Respective attached and suspension time points are indicated. Heatmap generated by clustering analysis of DEGs across samples. The Log2 fold changes of each gene were clustered based on euclidean distance. (D). Heatmaps for the individual OV90 samples using GSVA normalized enrichment scores (NES) for passage 0 (P0A-C), 1 (P1A-C), 3 (P3A-C), 4 (P4A-C), 6 (P6A-C), and 7 (P7A-C). The hallmarks were clustered based on euclidean distance. (E) Scatterplot showing the NES for samples on a 2-dimensional epithelial mesenchymal plane. (F) Scatterplot showing the NES for apoptosis and the NES for Oxidative Phosphorylation across time points for each sample in the replicates for OV90. (G) Scatterplot showing the NES for apoptosis and the NES for MYC Targets V2 for each time point for OV90.

We next assessed the transcriptional changes in response to suspension stress and during recovery periods of attached growth, to determine when most of the changes occurred in the adaptation process and what those changes indicate. Bulk RNA sequencing on OV90 or CAOV3 cells undergoing adaptation at various suspension and recovery time points revealed that the number of genes either upregulated or downregulated after the first exposure to suspension stress (P0 versus P1 after 24 hrs in suspension) was 1,011 for OV90 and 362 for CAV03 (DEGs, P0 vs P1, padj <= 0.05, L2FC >1.5) of which a total of 148 were upregulated and 863 downregulated for OV90 and 171 and 191 for CAOV3 respectively (Fig 4A–C, Supp 4A–C). In the case of OV90, 74 of the 148 upregulated genes were uniquely upregulated during the first exposure to stress (P0-P1) (Fig 4A). As cells adapted to stress in subsequent cycles, the total number of differentially expressed genes (DEGs) decreased over time, with more genes downregulated than upregulated in both OV90 and CAOV3 cell lines (Fig 4A–B, Supp 4A–B). The highest number of unique upregulated genes was observed from P0 to P7 (208 genes, OV90), and during the transition to resistance in OV90 cells (P0 and P3 time points ;165 genes). Most unique downregulated genes were found between P0 vs P7 (suspension AnR)) and P0 vs P6 (attached AnR), with 375 and 422 genes, respectively. 225 of these genes were consistently downregulated over time (Fig 4B last column). For CAOV3 as well, the comparison between P0 and P6 (2D comparisons of AnS and AnR), had a total of 131 up regulated genes and 213 down regulated genes, there were 29 unique up regulated genes, and 115 unique down regulated genes (Supp 4B–C). The P0 vs P7 comparison, as the time point with the greatest number of uniquely up and down regulated genes (110 and 379, respectively) in CAOV3 as well (Supp 4A–C) similar to OV90. While the number of genes per time point varied between the cell lines (Fig 4A–C, Supp 4A–C) the number of differentially downregulated genes exceeded the number of differentially up regulated genes in both models. Together these data suggest significant transcriptional changes occurring after the first exposure to stress, with only a subset of the same genes maintained in later stages of adaptation indicating changes in transcriptional programs over time.

Gene Set Variant Analysis (GSVA) of the different points using the Human MSigDB Hallmark Collections revealed a subset of hallmarks in OV90 cells that were primarily positively enriched early (before complete resistance) during the adaptation such as those associated with ‘ TGF beta signaling’ ‘ Hedgehog signaling’ and TNFa signaling via NFkb’ (Fig 4D). As anticipated with anoikis resistance, later passages showed reduced apoptosis (Fig 4D,F). Cells seemed to alternate between epithelial and mesenchymal states in OV90 cells, converging towards a hybrid EM state once adapted (Fig 4E). Some pathways were also more specific to the attached growth conditions of adapted cells such as mTORC1, Notch and Wnt beta catenin (Fig 4D, P6). GSVA analysis in a second cell line, CAOV3, identified distinct and overlapping enrichments with OV90 cells. Hallmarks including ‘TGF-β signaling’ ‘ Hypoxia’ and ‘Hedgehog signaling’ and TNFa signaling via NFkb that were more enriched in the early stages of the adaptation in OV90 remained enriched in CAOV3 even at later time points (Supp Fig 4D) suggesting some differences in timing of the changes of the pathways. Both cell lines however showed common enrichments in ‘Oxidative Phosphorylation’ and ‘MYC Targets’ in adapted cells (Fig 4F,G, Supp Fig 4D). Notably, the increase in MYC RNA levels (Supp Fig 4E i) was also manifesatd at the protein level (Supp Fig 4E ii). Lowering MYC in the adapted AnR OV90 cells using siRNA lowered the survival in suspension of the anoikis adapted (AnR) cells in vitro (Supp Fig 4F i,ii). These data together suggest common mechanisms enriched in adapted cells, with additional pathways/mechsnisms that may be cell line dependent and altered at different times during the adaptation. Notable changes in MYC in the adapted AnR populations directly contributed to anoikis resistance in the adapted population.

### Adapted anoikis resistant cells depend on oxidative phosphorylation for survival.

**Figure 5.**
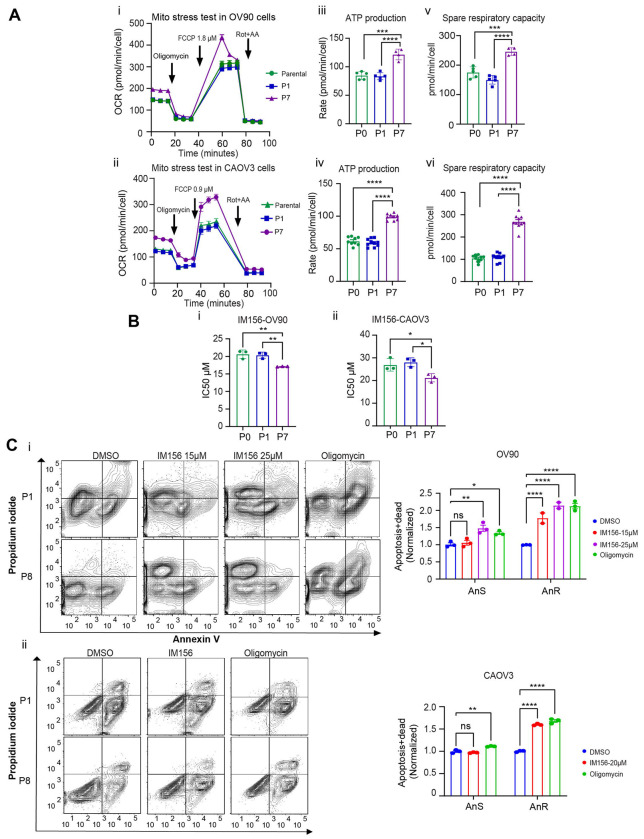
Adapted anoikis resistant cells are dependent on oxidative phosphorylation for enhanced survival upon loss of attachment (A). (i, ii) Representative Oxygen consumption rate (OCR) of cells from either parental (P0), cells exposed to one cycle of suspension culture (P1) or adapted AnR (P7) for indicated OV90 and CAOV3 cells measured using Seahorse XF96 mito stress test under attached conditions at baseline or after addition of Oligomycin (1.5 μm), FCCP (1.8 μm for OV90 and 0.9 μm for CAOV3), and Rotenone-Antimycin A (0.5μm) (iii, iv) ATP production and (v, iv) spare respiratory capacity of indicated cells calculated from i, ii. (n= 2). Data are normalized using SRB and Data are mean ± SEM. ns p > 0.05, *** p < 0.001, **** p <0.0001. One-way ANOVA followed by Tukey’s multiple comparison. (B). IC50 of (i) OV90 or (ii) CAOV3 cells to IM156 determined under attached conditions after 96 hrs using an SRB assay for cells from the indicated passages (n=3). Data are Mean ± SEM. ns p > 0.05, * p < 0.05, ** p < 0.01, One-way ANOVA followed by Tukey’s multiple comparison. (C) Flow cytometry analysis and quantitation (adjacent panel) of the apoptotic and dead cell population determined using Annexin V and propidium iodide staining of cells from indicated passages of either (i) OV90 or (ii) CAOV3 treated for 72 hours with DMSO, IM156, and Oligomycin in ultra-low attachment plates. Data are normalized to the respective DMSO controls (n= 3). Data are Mean ± SEM. ns p > 0.05, * p < 0.05, ** p < 0.01,**** p <0.0001, two-way ANOVA followed by Tukey’s multiple comparison.

Changes in the ‘Oxidative Phosphorylation’ pathway in adapted anoikis resistant cells was a hallmark in both cell lines tested despite their different mutational backgrounds (OV90 and CAOV3; depmap.org). We thus tested for a direct role for mitochondrial respiration in anoikis adapted cells. We first measured parameters of mitochondrial function by assessing respiration using extracellular flux analysis with a mitochondrial stress test (seahorse XF96 assay) in both OV90 and CAOV3 cells (Fig 5A i, ii). When comparing parental cells (P0) to cells after exposure to one round of suspension stress (P1), or to adapted AnR/P7 cells expanded in attached conditions, we find that AnR cells from both OV90 and CAOV3 cells exhibited higher oxygen consumption and extracellular acidification rates (OCR and ECAR respectively) at baseline compared to parental cells (P0) and cells from P1 (Supp Fig 5). ATP-dependent OCR, measured by adding oligomycin to inhibit Complex V (ATP synthase) of the electron transport chain (ETC) to the culture (Fig 5A i,ii), revealed significantly higher ATP-dependent OCR in P7/AnR cells (mean=121.5 pmol/min/cell −/+) compared to parental P0 cells (84.8 pmol/min/cell −/+) and P1 (83.4 pmol/min/cell −/+) in OV90 cells (Fig 5A iii). In CAOV3 cells, ATP-dependent OCR in P7 was also higher compared to P0 and P1 (Mean ATP production in Parental, P1 and P7 was 61.8, 59.2, and 98.9 pmol/min/cell respectively) (Fig 5A iv). Spare respiratory capacity (SRC) is a parameter representative of the mitochondrial ability to produce energy through respiration beyond the amount needed for basal cellular maintenance and has been shown to be increased during malignant transformation and tumor invasion. ^47^ SRC determined by adding FCCP (an uncoupler of mitochondrial oxidative phosphorylation) that shortcuts the ETC (Fig 5A) and increases OCR to its maximal level revealed that SRC was significantly higher in AnR/P7 cells compared to parental (P0) and P1 cells in both OV90 and CAOV3 models (Fig 5A v,vi).

Since adapted anoikis resistant cells showed higher SRC, we tested if in comparison to parental (P0) and P1 cells, AnR /P7 cells exhibited differential sensitivity to the biguanide IM156, an AMPK activator and Complex I inhibitor currently in clinical trials. ^48^ Remarkably, we found that adapted AnR cells from P7 for both OV90 and CAOV3 cells showed significantly lower IC50 to IM156 compared to parental and P1 cells. (OV90 mean IC50; parental= 20.6, P1= 20.3, P7=17.1μM and CAOV3 mean IC50; parental= 26.9, P1= 28, P7= 21.2 μM, Fig 5B i, ii). A sub IC50 dose of IM156 induced cell death in suspension (anoikis) to a significantly greater extent in the adapted AnR/P7 cells as compared to the parental counterpart (Fig 5C i,ii). Similarly, inhibition of the ETC complex V using oligomycin was able to resensitize and induce anoikis to a significantly higher degree in the adapted AnR P7 cells as compared to parental cells (Fig 5C i,ii). These data demonstrate that adapted anoikis resistant cells have developed an enhanced mitochondrial capacity to support their superior survival in suspension, and indicate that blocking OXPHOS in these cells could reverse such an adaptive phenotype.

### Evasion from immune surveillance by adapted anoikis resistant cells.

**Figure 6.**
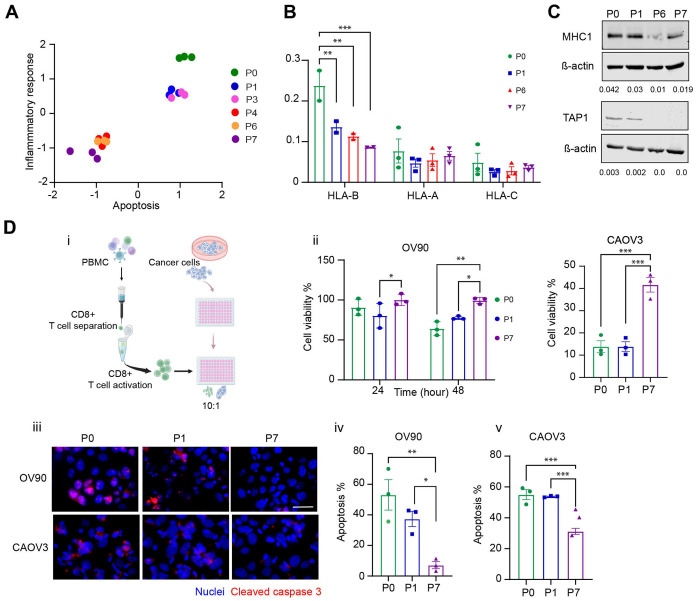
Adapted anoikis resistant cancer cells evade killing by cytotoxic T cells (A) Normalized Enrichment score (NES) for Hallmark inflammatory response pathway plotted against Hallmark apoptosis in OV90 cells derived from P0 (parental), P1, P3, P4, P6, and P7 (after 1 to 7 cycles of suspension culture for 24 hours respectively) n=3 replicates. (B) Semi qRT-PCR analysis of HLA-A, B, and C mRNA expression in indicated anoikis sensitive (P0, P1: after one round of 24 hr suspension stress) and isogenic anoikis resistant (P6, P7) OV90 cells. n=2-3 replicates. Data are mean ± SEM. ns p > 0.05 ** p < 0.01, *** p < 0.001. Two-way ANOVA followed by Tukey’s multiple comparison. (C) Representative western blot for TAP1 (lower) and MHC-I (upper) in indicated AnS and AnR derivative cells. Quantitation of TAP1 and MHC-I normalized to β-actin shown below. (n= 2). (D) Schematic of immune mediated tumor killing assay. CD8+ T cells were isolated from PBMCs and activated for 48 hours then co cultured with either OV90 or CAOV3 cells in the attached condition. (ii) Percent cell viability of indicated OV90 and CAOV3 cells measured by trypan blue staining after incubation with activated CD8+ T cells (24 and 48 hours for OV90 and 24 hours for CAOV3). n=3, Data are mean ± SEM. ns p > 0.05, * p < 0.05, ** p < 0.01, *** p < 0.001. Two-way ANOVA for OV90 and One-way ANOVA for CAOV3 followed by Tukey’s multiple comparison. (iii, iv, v) Representative widefield immunofluorescence images of Cleaved Caspase 3 (red) and DAPI (blue) (left), and quantitation of percent apoptosis (right) from indicated OV90 and CAOV3 cells co-cultured with activated CD8+ T cells (48 hours for OV90 and 12 hours for CAOV3 cells). Data are mean ± SEM. ns p > 0.05, * p < 0.05, ** p < 0.01, *** p < 0.001, **** p <0.0001, One-way ANOVA followed by Tukey’s multiple comparison. Scale bar: 50 μm. (n= 3 biological trials, each trial includes quantitation of a minimum of four 60X fields).

Bulk RNA seq results also revealed perturbation in pathways that may impact immune recognition of the adapted anoikis resistance cells compared with their sensitive counterpart. Of note, the inflammatory response pathway was enriched early during adaptation (P0 (parental), P1 and P3 (collected after 1 and 3 cycles of detachment respectively)) in comparison to later stages of adaptation P4, P6, and P7 (collected after 4,6, and 7 cycles of detachment respectively) in OV90 cells (Fig 6A). We also examined expression of HLA genes, as downregulation of MHC-I antigen presentation is a known mechanism of immune evasion in multiple cancers ^49^ and has been associated with anoikis resistance in other cancers. ^45^ We found that HLA-B as part of major histocompatibility complex I (MHC-I) but not HLA-A and C was downregulated over time during development of adaptive anoikis resistance in the attachment -detachment cycles in OV90 cells (Fig 6B). MHC-I protein expression in OV90 cells was also lower in adapted P6 and P7 compared with P0, P1 cells (Fig 6C). Additionally, TAP1 (Transporter associated with antigen processing 1) that is involved in MHC-I antigen presentation, was absent in adapted anoikis resistant cells (Fig 6C). TAP1 and MHC-I play crucial roles in tumor antigen recognition by Cytotoxic CD8+ T cells. ^50^ We thus hypothesized that adapted anoikis resistant cells that have reduced expression of MHC-I and TAP1 could be less susceptible to immune mediated killing compared to their anoikis sensitive counterparts. We isolated and activated CD8+ T cells from PBMCs derived from healthy individuals (Fig 6D) and added them to OV90 and CAOV3 cells from P0, P1, and P7 cultured in regular attached conditions (Fig 6D). We find a time dependent decrease in cell viability of the P0 OV90 (parental population) as compared to the adapted AnR OV90 cells (Fig 6D. ii). For CAOV3 cells we observed a much stronger and faster T cell response compared to OV90s, as at 24 hours, only 10 % viability was detected in the parental anoikis sensitive cells (P0, P1) while 40% of adapted anoikis resistant cells (P7) survived (Fig 6D. ii). To measure apoptosis of tumor cells induced by CD8+ T cells, we examined cleaved caspase 3 (CC3) after 48 hours of co culture of OV90 cells with T cells and 12 hrs of co-culture for CAOV3 cells due to their higher sensitivity (Fig 6D. ii). We find significantly higher CC3 in anoikis sensitive cells (P0, P1) as compared to the isogenic anoikis resistant cells (P7) (Fig 6D. iii–v). These data suggest that adaptation to detachment induced cell death in ovarian cancer cells faclitates immune evasion concomitant with reduced antigen presentation through downregulation of MHC-I and TAP1.

### Inhibiting the transcriptional Mediator kinase CDK8/19 prevented development of anoikis resistance in vitro and intraperitoneal growth in vivo

**Figure 7.**
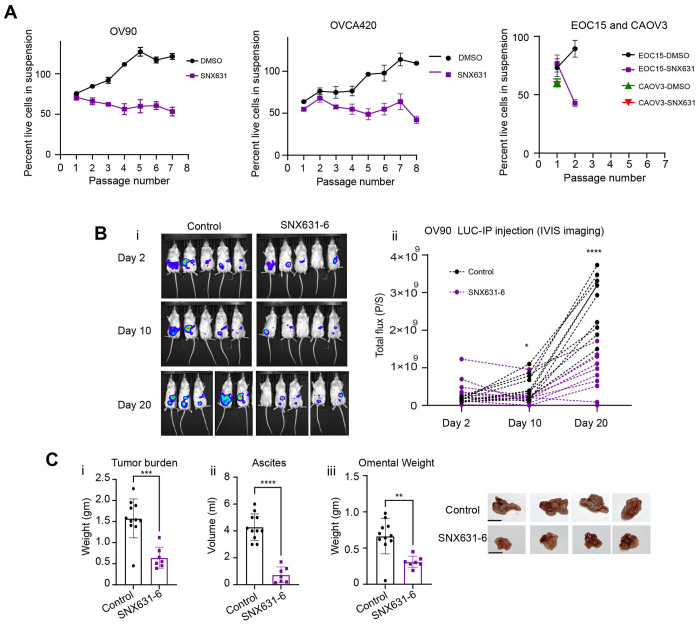
CDK8/19 Mediator kinase inhibition prevents development of anoikis resistance and ip xenograft tumor growth. (A) Percent survival of indicated anoikis sensitive cell lines (OV90, OVCA420, CAOV3, and EOC15) measured by trypan blue staining after 24 hours in suspension following cycles of gain and loss of attachment as in Fig 1B, either with vehicle (DMSO) or in the presence of 500nM CDK/19 Mediator kinase inhibitor (SNX631) (n= 3) (B). Whole body luminescence (BLI) (i) representative images of and (ii) quantification of total flux over time of NSG mice injected with 5 million live OV90 parental (P0) cells receiving either control diet or SNX631-6 medicated diet for the duration of the study. (n=11-12). Data are Mean ± SEM. ns p > 0.05, * p < 0.05, **** p <0.0001, two-way ANOVA followed by Tukey’s multiple comparison. (C) (i) Total tumor weight, (ii) ascites volume or (iii) omental weight, with example pictures (adjacent), from mice receiving either control diet or SNX631-6 medicated diet analyzed at end point on day 41. (n=7-11). Data are Mean ± SEM. ** p < 0.01, *** p < 0.001, **** p <0.0001 Student t-test.

Transcriptional changes led to pathways that were both overlapping and different between cell lines models including changes in the timing of the pathways being enriched. We thus tested the effects of preventing the overall process of transcriptional changes on development of adaptive anoikis resistance by inhibiting the kinase activity of RNA polymerase II transcription asssociated CDK8/19 Mediator kinases. For this, we used the selective inhibitor of CDK8/19 SNX631 ^51 52^ to test the effect on the development of anoikis resistance. We first assesed sensitivity of a panel of intrinsically anoikis sensitive cell lines OV90, CAOV3, EOC15, and OVCA420 to SNX631 over a course of 7 day treatments. We find the IC50s of the cell lines to range between 2.16 for OV90 to 4.1 for OVCA420 (Supp Fig 7A i,ii) which is over 2 orders of magnitide higher than the IC50 of SNX631 in a cell-based assay for CDK8/19 inhibition (11 nM).^51^ Hence, CDK8/19 activity is not required for the proliferation of the tested cell lines. Given the stress associated with matrix detachment, we chose a 500 nM concentration of SNX631 which does not inhibit cell growth but is sufficient for complete CDK8/19 inhibition and reducing the phosphorylation of STAT1 at S727 (Supp Fig 7A iii) a known substrate target of CDK8/19. ^53,54^ We found that treatment of OV90 and OVCA420 with 500 nM SNX631 during cyclic attachment -detachment culture cycles prevented development of anoikis resistance with mean percent live cells reaching a maximum of 53.28 % and 63.7 % in P7 respectively, while DMSO-treated control cells reached 100% or higher for both cell lines (Fig 7A) as seen in Fig 1. Two additional anoikis sensitive models CAOV3 and immortalized EOC15 cells were tested. 500nM SNX631 treatment in EOC15 and CAOV3 cells led to complete growth arrest of the cells following their first re-expansion in attached growth after a single 24 hr exposure to suspension cultures (Fig 7A). These data suggest that SNX631 prevented developing of AnR in some models and completely abrogated any expansion of cells in other. To assess whether global downregulation of the overall transcription rather than transcriptional reprogramming is sufficient to block the development of anoikis resistance we treated OV90 cells during cyclic culture of attachment -detachment with THZ1 which is a covalent CDK7 inhibitor that also targets CDK12 at higher doses and shown to globally downregulate transcription. ^55^ Interestingly, we observed that although a sublethal concentration (17nM) of THZ1 (chosen based on IC50 in OV90s Supp Fig 6B i) reduced the percent live cells at the endpoint mariginally (DMSO 108.4 %, THZ1 98.8% p value: 0.0344, Supp Fig 6Bii), this concentration of THZ1 was incapable of blocking the establishment of AnR in OV90 cells (Supp Fig 6B ii) suggesting selective dependence on CDK8/19 during development of anoikis resistance or the potential need of cytotoxic doses of THZ1 to be effective that is likely to impact other CDKs as well. ^55^

To further test whether CDK8/19 inhibition blocks intraperitoneal (ip) growth and metastasis of OV90 tumor cells, we randomized NSG mice into two groups: one received a regular diet and the other received SNX631-6 medicated chow (at 350 ppm) 1 day prior to injection of OV90 parental luciferase expressing cells into peritoneal cavity. Whole body bioluminescence imaging (BLI), revealed a significant increase in overall ip. tumor burden over time in mice receiving regular diet as compared to mice receiving the SNX631-6 diet (Fig 7B i,ii) We monitored BLI up to day 41 (Study endpoint) however the data is shown until day 20 (Fig 7B) as ascites build up in the control group starting at ~week 4 post tumor cell injection interfered with accurate luciferase detection in animals. Moreover, endpoint total tumor burden, ascites volume and omental tumor were significantly reduced in mice receiving SNX631-6 in their diet compared with mice receiving regular diet (Fig 7C i–iii). These results indicate that preventing the transcriptional changes is required to prevent the development of anoikis resistance and intraperitoneal tumor growth and metastasis in vitro and in vivo respectively.

## DISCUSSION

We report here that ovarian cancer cells exhibit a range of sensitivities to cell death upon matrix detachment in suspension. Cell line models that exhibit sensitivity can, when subjected to cycles of detachment and reattachment in vitro, acquire anoikis resistance. Such acquired anoikis resistance mimics the anoikis resistance after intraperitoneal ovarian cancer growth and tumor cell survival in ascites, but is not permanent, as cells can revert to their baseline state in the absence of detachment stress. Importantly, the acquired resistance is sufficient to increase migratory capabilities, resistance to chemotherapy, and confer the ability of the cells to metastasize readily to the lungs. Transcriptional reprogramming leading to changes in mitochondrial capacity and dependency and the ability to evade immune cell killing are some of the key phenotypic features of such cells. Critically, selective inhibitors of CDK8/19 Mediator kinase, a pleiotropic regulator of transcriptional reprogramming, prevent anoikis adaptation and metastasis in ovarian cancer models.

In ovarian cancer, the critical steps in metastasis include cell detachment from tubulo-ovarian regions and movement into the peritoneal cavity and circulation. Concomitant with metastasis, tumor cells evade apoptotic pathways upon loss of attachment. Disease spread and recurrence follow the reattachment and establishment of metastatic tumor colonies.^14,56,6,7,12^ Thus anoikis resistant cells and aggregates in ascites and in circulation (circulating tumor cells, CTC) have both been associated with poor clinical outcomes, specifically with relapse and chemoresistance.^57,58,2,59–62^ CTCs are thought to remain in circulation for several hours as the larger size of tumor cells compared to the small diameter of capillary vessels can lead to their arrest inside the small vessels of organs. ^61^ In the case of intraperitoneal metastasis cells typically perish due to hemodynamic forces, anoikis, or immune system attack, or dwell for a short time, as within 24 hours most cells are found attached to their secondary sites. ^35^ Interestingly, whole exome sequencing has revealed that the majority of mutations found in recurrent ascitic tumors were already present initially, ^63^ indicating that recurrence may stem more from adaptation rather than new mutations. Considering these notions, we exposed anoikis sensitive ovarian cancer cell lines to intermittent (24 hours) detachment induced stress followed by attachment intervals. Cells exposed to attachment detachment cycles gradually improved their survival under stress conditions (reduced apoptosis), which was not a consequence of enhanced proliferation or clonal selection**.** Each clone derived from a single cell at any time could in fact either be anoikis sensitive or resistant, and switch between these states**.** If a single cell was stably sensitive or resistant (two states in the population), we would have expected to observe a fixed survival of each clone under detachment in repeated trials over time. However, a spectrum of the percent survival in clones was observed. Additionally, the survival capacity of each clone fluctuated over time, indicative of the cells’ ability to interchange between sensitive and resistant states and also suggestive of non-mutational perturbation in cells, which was confirmed by whole exome sequencing. Our reversion experiments conducted under the absence of detachment stress revealed that the acquired resistance was indeed not permanent and hence likely non-genetic in nature. Hence, the findings herein were not concordant with two states and suggest likely additional states in the population. The Coefficient of Variation (CV) analysis implies the existence of either a pre-established cellular memory or the presence of multiple cellular states within the population. This diversity in cellular states results in varied responses to detachment stress, leading to greater differences in behavior among individual clones compared to a more uniform control group. In essence, it indicates that cells within the population respond differently to stress due to inherent or developed diversity, causing more variation in response, compared to a more homogenous control population. These hypotheses warrant future investigation and are important to our understanding of adaptation to biological stress inn physiology and disease.

A central question that we pursued was whether a non-permanent anoikis resistant phenotype was sufficient for metastatic capabilities. Indeed, much like prior studies on the concomitant increases in migration and resistance to anoikis, ^64^ adapted anoikis resistant cells were also more motile. Indeed, these two phenotypes are reciprocally related as forced confined migration can also conversely lead to anoikis resistance. ^45^ Chemosensitivity changes were consistent across several cell lines but were specific to paclitaxel and not other standard of care agents used for the management of ovarian cancer. MYC has been identified as a driver of paclitaxel resistance ^43,65,66^ and could indeed be a potential mechanism of paclitaxel resistance in the adapted anoikis cells that we found to have higher c-MYC mRNA and protein expression. Changes in mitochondrial activity of paclitaxel resistant cells have been reported previously in lung cancer, wherein paclitaxel resistant cancer cells had higher basal OCR and ECAR compared with sensitive cells. ^67^ Consistent with these previous studies, the adapted anoikis resistant cells not only consumed more oxygen/produced more ATP, but also had a higher spare respiratory capacity (SRC). We suggest that this enables the resistant cells to maintain their functions under an increased demand for energy under detachment conditions (stress stimuli). This manifested itself in higher sensitivity to OXPHOS inhibition, exposing a potential therapeutic vulnerability of adapted cells.

The common acquisition of metastatic phenotypes and similar changes in hallmark pathways during anoikis adaptation across two different ovarian cancer models indicate some common mechanisms of adaptation. However, distinct pathways were also uniquely altered in each model. CAOV3 cells are significantly more sensitive to matrix detachment stress compared to OV90 cells, and carry different mutations (depmap.org). It remains to be determined whether the inherent differences in baseline sensitivity to matrix detachment stress and/or the mutational backgrounds influenced the divergent adaptation pathways. Therefore, the differences in adaptation-associated pathway changes and their related dependencies may vary. This variability presents a therapeutic challenge, as manifested in the scarcity of targeted treatments universally applicable to the clinical management of ovarian cancer.

The dependency of anoikis adaptation on transcriptional changes was evident in its sensitivity to the inhibition of CDK8/19 Mediator kinases. CDK8 and CDK19 Mediator kinases, are alternative enzymatic components of the CDK module that regulates the transcriptional Mediator complex and are not part of the overall transcription machinery ^68^. CDK8/19 have been shown to regulate transcriptional reprogramming, acting as both positive and negative regulators of transcription. ^30^ Interestingly, many more genes were downregulated rather than upregulated during anoikis adaptation, which is consistent with the regulatory pattern of Mediator kinase, prolonged inhibition of which primarily upregulated rather than downregulated gene expression in 293 cells. ^30^ Of note, the steady state growth of ovarian cancer cells in vitro was only slightly affected by the selective CDK8/19 inhibitor SNX631 ^51^ as has also been observed in most other cancers, except for a subset of leukemias, and moderate responses in certain breast, colon, and prostate cancers. ^69–72^ In contrast, SNX631 effectively abrogated the development of anoikis resistance in various ovarian cancer models at a concentration (500 nM) that had little or no effect on ovarian cancer cell growth but was sufficient for nearly complete inhibition of CDK8/19 kinase activity. ^73^ The sensitivity of the anoikis adaptation process is aligned with prior studies where CDK8/19 inhibition prevented the development of drug resistance. ^51,74^

Transcriptional memory is a concept related to stress adaptation, where cells adjust their gene expression in response to environmental stimuli. ^75^ In our models, repeated exposure to stress might prime cells to modify their transcriptional programs more effectively upon re-exposure to similar stress. This enhanced response could then persist for several generations, even without continuous exposure to the initial stimuli ^75,76^ as seen in our memory studies. The role of CDK8 in transcriptional memory has been implicated in yeast and human cells, ^77,68^ and whether these mechanism are in play here remains to be determined. It is possible that most of the adapted cell phenotypes including the changes in EMT and mitochondrial dependencies and immune evasive characteristics would be abrogated by CDK8/19 inhibition. Prior studies using Cortistatin A (a different CDK8/19 inhibitor) showed changes in pathways involved in oxidative phosphorylation. ^78^ Similarly Senexin B (another CDK8/19 inhibitor) inhibited EMT in models including ovarian cancer. ^24^ Thus we anticipate that the effect of CDK8/19 inhibition is independent of the phenotypic outcomes, but rather central to the transcriptional adaptation to adversarial conditions. We propose that the lack of targeted therapies for advanced ovarian cancer is largely due to the extraordinary adaptability of cancer cells and their capacity for cellular reprogramming during intraperitoneal metastasis. Our findings implicate Mediator kinase paralogs, CDK19 and CDK8, which are general regulators of transcriptional reprogramming as a key dependency during development of anoikis resistance and thereby metastatic progression. This dependency on Mediator kinase presents a new therapeutic opportunity for the use of CDK8/19 inhibitors for the management of ovarian cancer in the clinic.

## MATERIALS AND METHODS

### Cell lines and culture conditions

Cell lines were cultured in their basal medium, 10% FBS, and 100 IU/ml penicillin 100μg/ml streptomycin unless indicated in Table 1 and were maintained in a 5% CO2 incubator at 37°C. Cell line authentication was done using STR profiling at the UAB Heflin Center for Genomic Sciences. Cells were tested frequently for mycoplasma contamination using LookOut^®^ Mycoplasma PCR Detection Kit (cat no: MP0035-1KT, Millipore sigma). CAOV3, P76, P151, P201, P211, HEK293 were cultured in DMEM (contains high glucose, L- glutamine and sodium pyruvate) supplemented with 10% fetal bovine serum (FBS). TYK-nu was cultured in DMEM+1% MEM non-essential amino acids+1% MEM vitamins. ID8-EMD, ID8-BRCA2, ID8-TP53 −/− were cultured in DMEM 4% FBS, 5 |ig/mL insulin, 5 μg/mL transferrin and 5 ng/mL sodium selenite. FT282 was cultured in 1:1 mixture of DMEM and F12 medium supplemented with 10% FBS and 2mM L- glutamine. OV90, EOC15 and IOSE141 were cultured in 1:1 mixture of MCDB 105 and Medium 199 supplemented with 15% and 10% FBS respectively. SK-OV3, OVCA433, OVCA420, HEY, HEYA8 and OVCAR10 were cultured in RPMI (contains l-glutamine) supplemented with 10% FBS and OVCAR3 with 20% FBS. OVCAR4 and OVCAR5 were cultured in RPMI supplemented with 10%FBS, 2mM glutamine, 0.25 U/mL insulin.

### Live cell/viability assessment of cell aggregates in suspension (multiple spheroids or cell aggregates)

6 well plates were coated with poly-HEMA (P3932-25G) (2% in 95% ethanol), then 1 ml poly-HEMA was added to each well and allowed to dry at 50° C overnight. The plates were UV-radiated for 1 hour. At this point, the plates were used for experiments or wrapped with Parafilm and kept at 4°C. Cells were cultured in 100mm plates until they reached ~90% confluency then trypsinized and counted with Trypan blue using Countess II FL Automated Cell Counter. 250,000 cells were cultured in poly-HEMA coated 6 well plates in full serum under regular growth medium (3 ml per well) for 24 hours. The cells from each well were collected in a 15 ml tube and centrifuged at 1200 rpm for 5 minutes. Next, the medium was removed and 100 μl of 10x Trypsin-EDTA (T4174-100ML Millipore sigma) was added to the cells and incubated for 5-20 minutes (depending on cell line and spheroid compactness) in the incubator. 400 μl medium was added to the cell suspension and the number of live cells was counted with Trypan blue exclusion using Countess II FL Automated Cell Counter.

### Live cell/viability assessment of single spheroids in suspension (single spheroid)

To obtain single spheroids, 1,000 OV90 and 2,500 CAOV3 cells were transferred to Ultra low attachment U bottom black wall 96 well plates (Corning: 4515) in 200 ul medium. The plate was centrifuged at 1200 rpm for 2 minutes and then incubated for 24 hours. For live dead assays, the LIVE/DEAD^™^ Viability/Cytotoxicity Kit, for mammalian cell: L3224 was used. Briefly, green-fluorescent Calcein-A and red-fluorescent ethidium homodimer-1 were added to HBSS buffer (Hanks’ Balanced Salt Solution) or PBS at 4 and 8 μM respectively. Then 100 μl of the medium was removed from each well and 100 μl HBSS containing dyes were added slowly to each well to have a final concentration of 2 and 4 μM of Calcein and Ethidium. After incubating the cells for 90 minutes, washing was done 3 times very carefully to avoid disrupting the spheroids (100μl of the dye solution was removed, and 100 μl of HBSS was added). Imaging was done using a confocal microscope (Nikon TE2000 inverted) at UAB’s High Resolution Imaging Facility and images were analyzed in Image J.

### Cyclic cell culture

250,000 cells were cultured in poly-HEMA coated 6 well plates for 24 hours. Cell number and dish size were kept constant to obtain reproducible live cell numbers. After 24 hrs, the cells were centrifuged at 1200 rpm for 5 minutes and viability was measured as described under ‘ *Viability assessment of cell aggregates in suspension’* prior to proceeding to plating the cells into standard tissue culture 2D conditions in a 60mm TC dish until they reached 90% confluency. The media was changed every 3-4 days. The first three steps were repeated for another 6-8 cycles depending on the cell line (total 7-9 passages).

### Memory/reversion studies

Adapted anoikis resistant (AnR) OV90 and CAOV3 cells from suspension culture were seeded into 60mm dishes and sub cultured in 2D for a total of 9-11 passages in 60mm dishes. At each passage, when the cells became 80-90% confluent, a fraction of cells were cultured in suspension in polyhema-coated plates for 24 hours and live cell/viability was measured as described above under ‘ *Viability assessment of cell aggregates in suspension’*. The remaining cells were maintained in 2D. The number of generations for these experiments was calculated as the number of times the cell population doubled over time. The number of generations in each passage was calculated using: log2 (*x*) where x is equal to the (final cell number/initial cell number.)

### Doubling time (DT) measurement in 2D

5000 cells were cultured in a 96 well plate in 100 μl medium and SRB assay as described previously ^79^ and was done every 24-48 hours for 7 days. Absorbance was measured at 570 nm using a Synergy H1 microplate reader (Biotek). DT was calculated using absorbance values obtained from the growth phase of the cells using the equation: *DT = ln2*/((*ln* [*OD2/OD1*]) / (*T*2 – *T*1)). OD1 and OD2 are the absorbance values measured in Time 1 and 2 (T1, T2) respectively.

### Doubling time measurement in suspension

250,000 cells were cultured in suspension in a 6 well poly-HEMA coated plate. Cell viability was measured using a Trypan blue exclusion assay every 24-48 hours for 10 days. Doubling time (DT) was calculated using the equation: DT = ln2/((ln [CN1/CN2 ]) / (T2-T1)). CN1 and CN2 are the cell number values measured in Time 1 and 2 (T1, T2) respectively.

### Flow cytometry-based apoptosis assay.

Parental and AnR OV90 and CAOV3 cells were cultured in suspension for 24 hours as described above. The cells were treated with Trypsin −EDTA for 5-10 minutes to produce a single-cell suspension. Media was added to the cell suspension to neutralize trypsin. Then, 500,000-100,0000 cells were transferred to a new 1.5 microcentrifuge tube and washed with Cold PBS twice. The cells were stained with Annexin V and PI using (eBioscience^™^ Annexin V Apoptosis Detection Kits: 88-8005-74) according to the manufacturer’s protocol. Flow cytometry was done using BD LSRFortessa (Europa) at the UAB flow cytometry core. Analysis was carried out in FlowJo 10.8.1.

### Ki67 Immunofluorescence staining in suspension

1 million cells were collected in 15 ml tubes after 24 hours of suspension culture. The cells were centrifuged at 1200 rpm for 5 minutes and then transferred to 1.5μL microcentrifuge tubes. (All staining steps were done in 1.5 ml tubes as follows: (Note: between each step cells were centrifuged at 2000 rpm for 2 minutes) cells were washed in 400-μl cold PBS for 5 minutes twice then fixed in 400-μl freshly made 4% PFA pH:7.4 at RT for 15 minutes. Washing was done with 400 μl PBS once for 5 minutes. The cells were quenched with 400-μl, 10 mM NH4CL for 5 minutes. Washing was done with 400 μl PBS once for 5 minutes then cells were permeabilized in freshly made 400 μl, 0.3 Triton 100x (dissolved in PBS by sonication) for 10 minutes. Blocking was done by incubating the cells in 400 μl, 5% BSA in PBS for 1 hour at RT. Ki-67 (8D5) Mouse mAb #9449 antibody was diluted 1:450 in 3% BSA in PBS and 100 μl was added to the cells for overnight incubation at 4°c on a shaker. The next day, cells were washed in PBS thrice for 5 minutes and incubated in Goat anti-Mouse IgG1 Cross-Adsorbed Secondary antibody; Alexa Fluor^™^ 594 # A-21125 diluted 1:500 in 3% BSA in PBS for 1 hour at RT in dark. Washing was done 3 times in PBS for 5 minutes. To stain the nuclei, DAPI (10mg/ml) was diluted 1:2000 in PBS and added to the cells for 10 minutes. Cells were washed with PBS once and centrifuged. The cell pellet was suspended in 200 μl PBS and 100 μl of cell solution was cytospun on a microscope slide at 800 rpm for 5 minutes and mounted with ProLong^™^ Gold Antifade Mountant # P36930). Imaging was done using a confocal microscope (Nikon TE2000 inverted) and images were analyzed in Image J as follows: Corrected total cell Fluorescence (CTCF) was measured using the formula: Integrated density of Ki-67 in a certain field - (Surface area of the field × Mean background fluorescence). CTCF of Ki-67 was normalized to CTCF of DAPI in the same field.

### 3D Cell viability assessment in single clones

Single Cell cloning was carried out by serial dilution of OV90 cells in 96 well plates (Corning protocol) (https://www.corning.com/catalog/cls/documents/protocols/Single_cell_cloning_protocol.pdf). After 2 weeks, single clones were trypsinized and seeded in 6 well plates and passaged in attached conditions (2D) for 6 passages. The viability of each clone in suspension from passages 1,3, and 6 was analyzed using Trypan blue exclusion.

### Patient and mouse ascites derived cells

Cells from patient ascites were established as described by us previusly ^18,20^ with approval for the study granted from the Penn State College of Medicine and UAB Institutional Review Boards (IRB). 50 ml ovarian cancer patient-derived ascites fluid or mouse ascites fluid (variable volumes) collected after ip injection of human OV90 or ID8 cells as indicated in figures, was centrifuged at 4000 rpm for 20 minutes. RBC hemolysis buffer (8.29 g Ammonium chloride, 1.0 g potassium bicarbonate, and 0.037g EDTA Dissolved in 1 liter of Milli-Q water and autoclaved) was added to the cell pellet (10:1 v/v) and incubated at RT for 10 min. PBS was added to the cell suspension to stop the reaction (10x volume) then centrifugation was done at 300 g for 10 minutes. The pellet was suspended in pertinent media and mixed well. Cell counting was done by Trypan blue exclusion using Countess II FL Automated Cell Counter. EOC 15 primary cells were cultured in 6 well Poly Hema plates at 250k cells per well for 2 weeks with standard medium change every 4 days. After a week, spheroids were disaggregated and cultured in 2D on tissue culture treated plates. EOC 15 were subsequently maintained in 2D. To *immortalize* human primary cells (used in a subset of experiments as indicated in legends), 50,000 cells were seeded in 12 well plates. hTERT (LVP 1130-Puro-PBS) and SV40 large T-antigen (LVP016) lentivirus at MOI of 15 and Polybrene at 10μg/ml concentration were added to the culture and incubated for 24 hours then the medium was changed to fresh medium and incubated for 48 hours. To select the successfully immortalized cells Puromycin was added at 5μg/ml concentration to the culture and incubated for 48 hours.

### Immune mediated tumor killing assay

CD8+ T cells were isolated from normal healthy female PBMCs (Precision for Medicine) using Miltenyi Biotec, Inc. CD8+ T cells isolation kit (catalog no: 130-096-495) following the manufacturer’s protocol. The isolated T cells were cultured at a density of 500,000 cells per well of 48 well plate in RPMI medium supplemented with 10% FBS, 1% pen/strep, and 30 U/ml IL2 and incubated in CO2 incubator at 37c. 48 hours before starting the killing assay, T-cell activation was done using Dynabeads^®^ Human T-Activator CD3/CD28 (Gibco: 11161D) following the manufacturer’s protocol. Briefly, 1 million CD8+ T cells were resuspended in 500 μL fresh medium and 25 μL prewashed Dynabeads were added to the cell suspension and transferred to 24 well plates and incubated in CO2 incubator for 48 hours. 10000 parental, P1 and, P7 OV90 and CAOV3 cells were cultured in 96 well plate in their growth medium for 24 hours then Activated CD8+ T cells were suspended in tumor cells growth medium (Dynabeads should be removed) and added to the wells at a ratio of 10:1 (T cell/Tumor cells). The number of surviving tumor cells was counted after 24- and 48-hour incubation with immune cells using trypan blue exclusion assay (the wells were washed with medium 4-5 times to remove T cells before counting the tumor cells). To measure percent apoptosis in Tumor cells induced by T cells, OV90 and CAOV3 cells were cultured in a glass bottom 96 well plate as described and stained with Cleaved caspase 3 (CC3) antibody (Cell signaling technology: 9661T) at 1:100 dilution after 12 hours incubation with T cells for CAOV3 and 48 hours incubation for OV90 cells. To calculate the percent apoptosis, the number of positive cells for CC3 was counted using Image J and divided by the total number of cells in each field.

### SNX631 and THZ1 treatment in OV90 cells, OVCA420

The cells were cultured in TC-treated plates until 80% confluent. Following trypsinization, 250,000 cells were cultured in poly-HEMA coated 6 well plates in medium containing either DMSO, or indicated concentrations of SNX631 or THZ1. After 24 hours of incubation, live cell count/viability was measured using Trypan blue, and 500,000 cells were seeded in 60mm dishes in the presence of either DMSO, SNX631, or THZ1 until 80% confluent. This cycle of attachment-detachment (in the presence of drugs) continued as described in the figures.

### Oligomycin and IM156 treatment in OV90 and CAOV3 in suspension culture

250,000 OV90 or CAOV3 from either P0 (Parental) or P7 (undergone 7 cycles of attachment-detachment) were cultured in poly-HEMA coated 6 well plates in their respective growth media containing DMSO as the control and either 1.5 μM Oligomycin or IM156 at 20 μM for CAOV3, 15 and 25 μM respectively for OV90 cells and incubated for 72 hours. Next, the cells were collected and trypsinized in 10x Trypsin for 10 minutes to make single-cell suspensions, and cell death and apoptosis were measured by flow using annexin/PI staining.

### Seahorse Mitochondrial stress test

Seahorse Mito stress test was done following the UAB Bioanalytical Redox Biology (BARB) core XF96 assay protocol; 20000 OV90 and 25000 CAOV3 parental cells P0 (parental), P1 (1 cycle of detachment), and P7 (7 cycles of detachment) were cultured in 100 μl growth medium in XF96 cell culture microplates and incubated for 36 hours in CO2 incubator at 37 c. 1 day before the experiment, XF96 assay cartridge was hydrated in sterile water and placed in a non-CO2 incubator at 37 c. On the day of the experiment, water was replaced with pre-warmed calibrant and placed in a non-CO2 incubator for 60 minutes, meanwhile, the cell culture medium was removed from the microplate and cells were washed with pre-warmed XF assay media (DMEM with 10 mM glucose, 2 mM glutamine, and 1 mM pyruvate) twice and incubated in the same media for 1 hour at 37c in a non- CO2 incubator. Next, effectors were added to the cartridge ports as follows: Port A: Oligomycin 20 μl at final 1.5 μM concentration, Port B: FCCP: 22μl final concentration of either 0.675 or 0.9 μM, Port C: FCCP: 25 μL final concentration of either 1.65 or 1.8 μM, Port D: Antimycin a/Rotenone: 28 μl at final concentration of 0.5 μM each) and put into the XFe96 extracellular flux analyzer to equilibrate. After 1 hour of incubation, cells were washed with a final volume of 180 μl assay media in each well, and the assay was run as follows; Calibration and equilibration each for 15 minutes, Cycle; Mix for 3 minutes, wait for 0 minutes and measure for 3 minutes, Inject Port A Cycle; Mix for 3 minutes, wait for 0 minute and measure for 3 minutes, Inject Port B; Cycle; Mix for 3 minutes, wait for 0 minute and measure for 3 minutes, Inject port C; Cycles; Mix for 3 minutes, wait for 0 minute and measure for 3 minutes, Inject port D; Cycle; Mix for 3 minutes, wait for 0 minute and measure for 3 minutes. Analysis was done using Seahorse Wave 2.6 and GraphPad Prism 10.0.2.

### IC50 determination

Ovarian cancer cells were seeded in 96 well plates at indicated cell density/well (Table 1) and incubated until 50-60 % confluent. Cell growth medium containing drugs (Cisplatin, Doxorubicin, Paclitaxel, IM156, SNX631, or THZ1) was added in 8 dilutions to the cells and incubated for 48 hours to 7 days followed by SRB assay. Absorbance was measured at 570 nm using a Synergy H1 microplate reader (Biotek). IC50 was calculated using nonlinear regression (four parameters) in GraphPad Prism software 10.0.2.

### Transwell fibronectin migration

24 well transwell with 8 p,m pore membrane (Greiner bio-one, catalog: 662638) was coated with 10 μg/ml fibronectin in sterile DI water solution and incubated at 37° C for 2 hours. Then cells were suspended in 100 μl serum-free medium and added to the upper chamber of each transwell (refer to Table 2 for cell number and incubation time). The lower chamber was filled with 600 μl Full serum medium and it was incubated in a CO2 incubator at 37° C for indicated hours. Then the medium was removed and transwells were washed with PBS twice. Non-invaded cells were scraped by a cotton swab. Then cells were fixed with 4% PFA (paraformaldehyde, Avantor, catalog: S898-07) at RT for 5 minutes. Cells were permeabilized with 100 % ethanol for 20 minutes. Next, the cells were stained with 0.5% Crystal violet (Alfa Aesar catalog: B21932-22) for 15 minutes and washed with DI water. Transwells were air dried at RT overnight and membranes were cut and mounted on glass slides with Permount (Fisher scientific catalog: S70104). Imaging was done using EVOS M7000 inverted microscope (Thermo fisher).

### Animal studies

All animal studies were performed in accordance with the Institutional Animal Care and Use Committee at the University of Alabama Birmingham. Female NSG or SCID mice from Jackson labs 5–8 weeks of age were housed under pathogen-free conditions at the Animal Research Facility at UAB. Tumor growth comparison in parental and adapted anoikis resistant cells: For OV90 cells, 8-week-old NOD.Cg-Prkdcscid/J (SCID, Jackson labs) mice were randomized. OV90 LUC GFP cells derived from P0 (Parental), and P7 (7 cycles of detachment) were expanded in TC treated plates. Subsequently, 5 × 10^6^ were injected intraperitoneally. Tumor progression and disease burden was tracked every 10 days by IVIS Lumina III *In vivo* Imaging System (Caliper Life Sciences, MA) at UAB’s Small Animal Imaging Facility. Mice were euthanized between day 39-40 when they became moribund. Necropsy was done and lung tissue was transferred to 24 well plates with 1 ml medium containing 150 μg/ml D-Luciferin and incubated at 37°C for 10 minutes and bioluminescence imaging was conducted using IVIS Lumina II. Ascites fluid volume and total tumor weight was measured at the endpoint and collected for further analysis. For ID8 cells, 8-week-old C57BL/6J mice were randomized. Parental ID8-EMD cells P0 (parental) or P7 (7 cycles of detachment) were expanded in TC-treated plates and 10 × 10^6^ cells were injected intraperitoneally. Weight and girth measurement was done every 10 days and mice were euthanized after 11.5 weeks. Necropsy was done and ascites fluid volume and tumor weight were measured and collected.

#### SNX631 study:

Twenty-four 8 weeks old NOD.Cg-Prkdcscid Il2rgtm1Wjl/SzJ (NSG from Jackson labs) mice were randomized into 2 groups receiving either control or SNX631-6 medicated diet (350 ppm, providing a daily dose of 30–50 mg/kg, Senex Biotchnology). 5 × 10^6^ OV90 LUC GFP cells were injected intraperitoneally (ip). Animals were observed daily and tumor progression and disease burden were tracked every 10 days by IVIS Lumina III imaging System (Caliper Life Sciences, MA) at UAB’s Small Animal Imaging Facility along with weight and girth measurements every 10 days. Mice were euthanized on day 41 when they became moribund. Necropsy was done and ascites fluid volume and total tumor weight were measured.

### Bulk RNA-seq analysis (secondary analysis and differential gene expression)

Total RNA was isolated using Trizol/Chloroform extraction method and RNA quality was validated on RNA-1000 chip using Bioanalyzer (Agilent). 1.0 ug of total RNA was used for the construction of sequencing libraries. For library preparation, Novogene utilized the NEBNext UltraTM RNA Library Prep Kit for Illumina on an Illumina NovaSeq 6000. All samples contained a minimum of 24 million reads with an average number of 29.2 million reads across all biological replicates. FASTQ files were uploaded to the UAB High-Performance Computer cluster for secondary analysis with the following custom pipeline built in the Snakemake workflow system (v5.9.1)^1^: first, quality and control of the reads were assessed using FastQC, and trimming of the bases with quality scores of less than 20 and adapter were performed with Trim_Galore! (v0.6.4). Following trimming, read quality was re-assessed with FastQC and splice-aware mapping was performed with STAR^2^ (v2.6.0c, with ‘2-pass‘ mode) using the GENCODE GRCh38 primary assembly and annotation GTF (release 37). Following genome mapping, BAM index files were generated with SAMtools^3^ (v1.9) and quality control of aligned files was performed with RSeQC^4^ (v3.0.1). Lastly, count generation was performed with ‘featurecounts‘ (with ‘Rsubread‘^5^, v.1.32.2 and R v3.5.1) and logs of reports were summarized and visualized using MultiQC^6^ (v1.6). Tertiary analysis was performed in R (v 4.0.2) with the DESeq2 ^7^ package (v1.34.0). Briefly, pre-filtering of low abundance genes was performed to keep genes that have a mean of at least 5 counts, and normalization was performed. Following count normalization, principal component analysis (PCA) was performed, and genes were defined as differentially expressed genes (DEGs) if they passed a statistical cutoff containing an adjusted p-value <0.05 (Benjamini-Hochberg False Discovery Rate (FDR) method) and if they contained an absolute log_2_ fold change >=1). Additional Pathway analysis was performed using GSVA 1.42.0 ^80^ utilizing the Hallmarks from the Human MSigDB Collections. The gene signatures for epithelial and mesenchymal gene sets were obtained from. ^81^ Heatmaps were made with ComplexHeatmap version 2.10.0 ^82^ with the normalized enrichment score from GSVA using the Euclidean distance method. Hallmark scatterplot graphs use normalized enrichment scores from GSEA with ClusterProfiler and were plotted using GraphPad Prism. The FASTQ files of the current study have been uploaded to NCBI’s Gene Expression Omnibus under accession number GSE241546. Reference for R studio: Posit team (2023). RStudio: Integrated Development Environment for R. Posit Software, PBC, Boston, MA. URL http://www.posit.co/. UpSet plots were made using ComplexUpset R package v 1.3.3. https://cran.r-project.org/web/packages/ComplexUpset/index.html and ^83^

### Whole Exome Analysis

Isolation of DNA was performed using Qiagen DNeasy Blood and Tissue Kit. WES library preparation and sequencing were performed by Novogene utilizing Agilent SureSelect Human All Exon V6 Kit yielding paired-end 150-bp reads on an Illumina NovaSeq 6000 and a median coverage of >159X, for the parental cells P0 and the adapted P7 cells using OV90. Results were provided as demultiplexed paired FASTQs containing the sequencing reads. Sequencing was targeted to a mean depth of at least 100x mean coverage in capture regions. Sequence alignment, quality control, and variant calling were done following GATK best practices using the germline and somatic variant calling algorithms haplotype caller and mutect2, respectively. All sequencing had an alignment of 99.8-99.9%. MAFtools version 2.10.05 ^84^was used to compare the sequenced time points. Code Availability: https://github.com/page22emily/RNAseq_Anoikis

### Semi quantitative RT-qPCR

Total RNA was harvested using Trizol/Chloroform extraction. RNA was transcribed using iScript Reverse Transcription Supermix and iTaq Universal SYBT Green Supermix. Expression data was normalized to RPL13A or HPRT or GAPDH. RT-qPCR primer sequences are listed in Table 4.

## Supplementary Material

Supplement 1

## Figures and Tables

**Table 1:** 

Cell line	Initial cell density
OV90	5000
OVCA420	2000
CAOV3	5000
EOC15	5000

**Table 2:** 

Cell line	Initial cell density	Incubation time (hours)
OV90	100000	24
CAOV3	100000	24
OVCA420	75000	24
EOC15	100000	24
P151	75000	6

**Table 3.** Resources

REAGENT	SOURCE	IDENTIFIER
**Cell lines**		
OV90	ATCC	CRL-11732
CAOV3	ATCC	HTB-75
OVCAR3	NIH	NCI60 (0507709)
OVCAR4	NIH	NCI60
OVCAR5	NIH	NCI60
OVCAR10	Susan Murphy (Duke University)	
SK-OV3	ATCC	bnHTB-77
OVCA433	Susan Murphy	N/A
OVCA420	Susan Murphy	N/A
HEY	Susan Murphy	N/A
HEYA8	Susan Murphy	
TYK-nu	Susan Murphy	
P76	Amir Jazaeri	N/A
P151	Amir Jazaeri	N/A
P201	Amir Jazaeri	N/A
P211	Amir Jazaeri	N/A
FT282	ATCC (Ronny Drapkin)	
**IOSE141**	Canadian Tissue bank	
ID8-EMD	EMD Millipore	SCC145
ID8-Trp53 −/−	Ian McNeish	
HEK293	ATCC	CRL-1573
EOC15	Penn state/UAB	N/A
**Commercial Kits**		
LIVE/DEAD^™^ Viability/Cytotoxic kit	Fisher Scientific	L3224
LookOut^®^ Mycoplasma PCR Detection Kit	Millipore sigma	MP0035-1KT
Annexin V Apoptosis Detection Kits	eBioscience^™^	88-8005-74
CD8+ T cells isolation kit	Miltenyi Biotec	130-096-495
DNeasy Blood and Tissue Kit	Qiagen	69504
**Antibodies**		
Ki-67 (8D5) Mouse mAb	Cell signaling	9449
Goat anti-Mouse IgG1 Cross-Adsorbed Secondary antibody Alexa Fluor^™^	Invitrogen	A-21125
Cleaved caspase 3	Cell signaling technology	9661T
YH2AX	Cell signaling technology	9718T
annexin V	Bio Legend	640906
PI	Invitrogen	00-6990-42
pSTAT1	Cell signaling technology	9177S
MHC1	R&D	NBP3-16696
TAP1	Cell signaling technology	49671S
MYC	Cell signaling technology	13987
**Other reagents and recombinant constructs**		
ProLong^™^ Gold Antifade Mountant	Invitrogen	P36930
Dynabeads^®^ Human T-Activator CD3/CD28	Gibco	11161D
Crystal violet	Alfa Aesar	B21932-22
Permount	Fisher scientific	S70104)
Oligomycin	Sigma Aldrich	O4876-5MG
IM156	MedChem Express	HY-136093A
THZ1	MedChem Express	
SNX631	Senex Biotechnology	
SNX631 (Diet)	Senex Biotechnology	
Poly-HEMA	Sigma Aldrich	P3932-25G
hTERT	Gen Target	LVP1130-Puro-PBS
SV40 large T-antigen lentivirus	Gen Target	LVP016-Hygro
siC-MYC	Thermo fisher	4392420 (s9130)
siC-MYC	Thermo fisher	4392420 (s9129)
siNTC	Thermo fisher	4390843
**Software used**		
ImageJ	NIH	https://imagej.net/ImageJ
Prism9	GraphPad	https://www.graphpad.com/scientific-software/prism/
Adobe Illustrator	Adobe	https://www.adobe.com/products/illustrator.html

**Table 4.** qRT-PCR Primers (listed 5’ to 3’)

1	RPL13A F human: GGC CCA GCA GTA CCT GTT TA
	RPL13A R human: AGA TGG CGG AGG TGC AG

2	HLA-A F human: AGA TAC ACC TGC CAT GTG CAG C
	HLA-A R human: GAT CAC AGC TCC AAG GAG AAC C

3	HLA-B F human: CTG CTG TGA TGT GTA GGA GGA AG
	HLA-B R human: GCT GTG AGA GAC ACA TCA GAG C

4	HLA-C F human: GGA GAC ACA GAA GTA CAA GCG C
	HLA-C R human: ACA TCC TCT GGA GGG TGT GAG A
