# Supplementary material for: Development of adaptive anoikis resistance promotes metastasis that can be overcome by CDK8/19 Mediator kinase inhibition
Source: bioRxiv. 2023 Dec 6:2023.12.04.569970. Preprint. [Version 1] doi: 10.1101/2023.12.04.569970 (PMC10723298; doi:10.1101/2023.12.04.569970)
Supplement: Supplement 1 [file media-1.pdf]

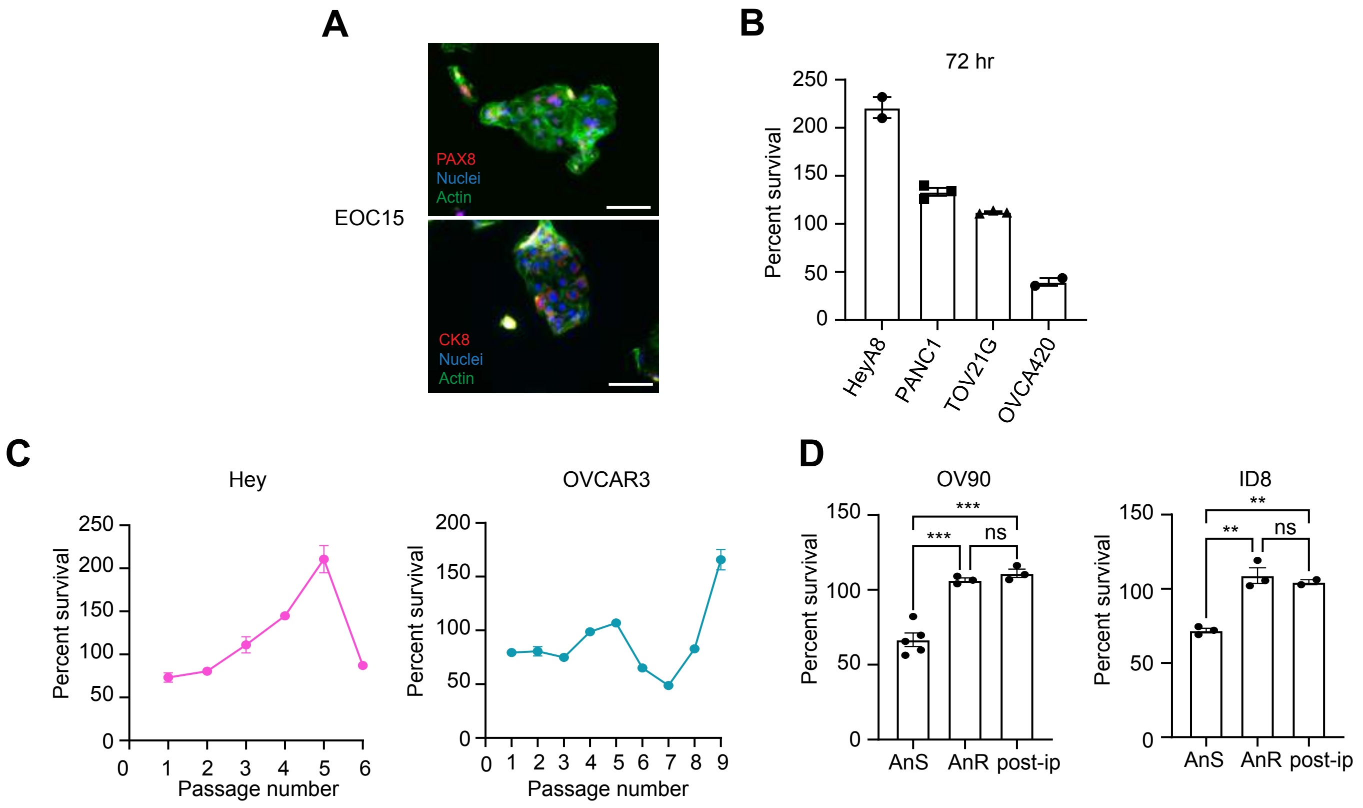
A
PAX8
Nuclei
Actin
EOC15
CK8
Nuclei
Actin
B
72 hr
Percent survival
250
200
150
100
50
0
HeyA8
PANC1
TOV21G
OVCA420
C
Hey
Percent survival
250
200
150
100
50
0
0 1 2 3 4 5 6
Passage number
OVCAR3
Percent survival
200
150
100
50
0
0 1 2 3 4 5 6 7 8 9
Passage number
D
OV90
Percent survival
150
100
50
0
***
***
ns
AnS
AnR
post-ip
ID8
Percent survival
150
100
50
0
**
**
ns
AnS
AnR
post-ip

Supp Figure 1:

(A) Representative immunofluorescence images of F-actin (green), PAX8, and CK8 (red) from EOC15 primary cells, Scale bar: 100 µm. (B) Percent live cells in suspension of indicated cell lines- HeyA8, PANC1, TOV21G and OVCA420 cells measured by trypan blue staining following 72 hours incubation in suspension culture (n= 2-3 replicates/cell line). (C) Percent live cells in suspension of Hey (left) and OVCAR3 (right) cells following cyclic model of gain and loss of attachment from Fig 1B. Live cells were measured by trypan blue staining after 24 hours in suspension and plotted as percent relative to initial plating number (n= 2-3 replicates). (D) Percent live cells in suspension of OV90 (left) and ID8 cells (right) derived from P0 (Parental, AnS), P7 (after 7 cycles of detachment, AnR) compared with the cells derived from ascites fluid at end point of mice that received OV90 and ID8 cells intraperitoneally. Cell survival was measured by trypan blue staining after 24 hours in suspension and plotted as percent survival in suspension (n= 2-5 replicates). Data are mean ± SEM. ns $p > 0.05$, * $p < 0.05$, ** $p < 0.01$, *** $p < 0.001$, Two-way ANOVA followed by Tukey's multiple comparison.

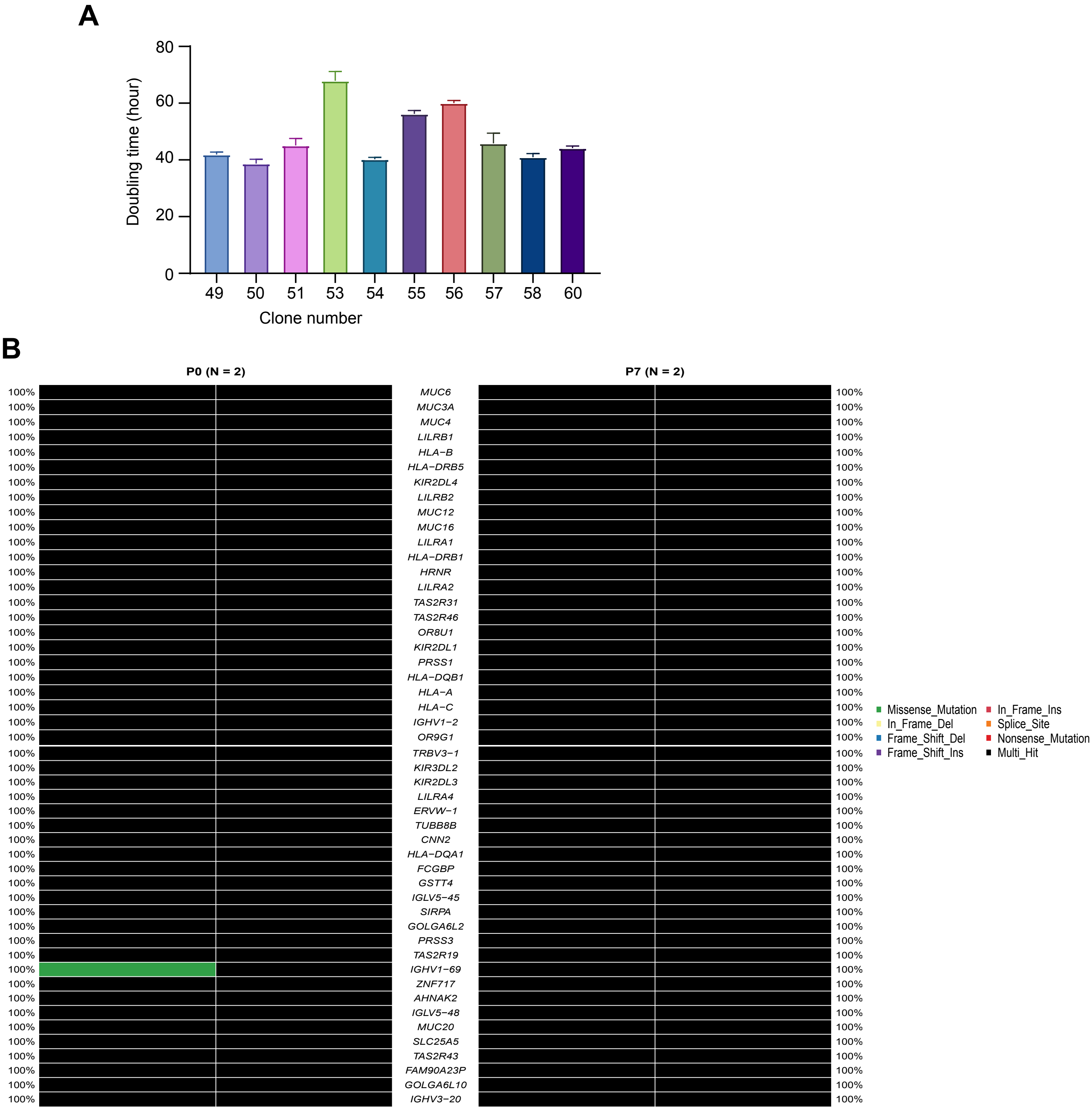
A
Doubling time (hour)
0
20
40
60
80
49
50
51
53
54
55
56
57
58
60
Clone number
B
P0 (N = 2)
P7 (N = 2)
MUC6
MUC3A
MUC4
LILRB1
HLA−B
HLA−DRB5
KIR2DL4
LILRB2
MUC12
MUC16
LILRA1
HLA−DRB1
HRNR
LILRA2
TAS2R31
TAS2R46
OR8U1
KIR2DL1
PRSS1
HLA−DQB1
HLA−A
HLA−C
IGHV1−2
OR9G1
TRBV3−1
KIR3DL2
KIR2DL3
LILRA4
ERVW−1
TUBB8B
CNN2
HLA−DQA1
FCGBP
GSTT4
IGLV5−45
SIRPA
GOLGA6L2
PRSS3
TAS2R19
IGHV1−69
ZNF717
AHNAK2
IGLV5−48
MUC20
SLC25A5
TAS2R43
FAM90A23P
GOLGA6L10
IGHV3−20
100%
Missense_Mutation
In_Frame_Del
Frame_Shift_Del
Frame_Shift_Ins
In_Frame_Ins
Splice_Site
Nonsense_Mutation
Multi_Hit

Supp Figure 2:

(A). Doubling time of indicated clones (randomly chosen) measured by an SRB assay every 24-48 hours over a 7-day period. (B). Oncoplot of the top 50 mutated genes based on the total number of mutations present across 2 time points, P0 (left) and P7 (right).

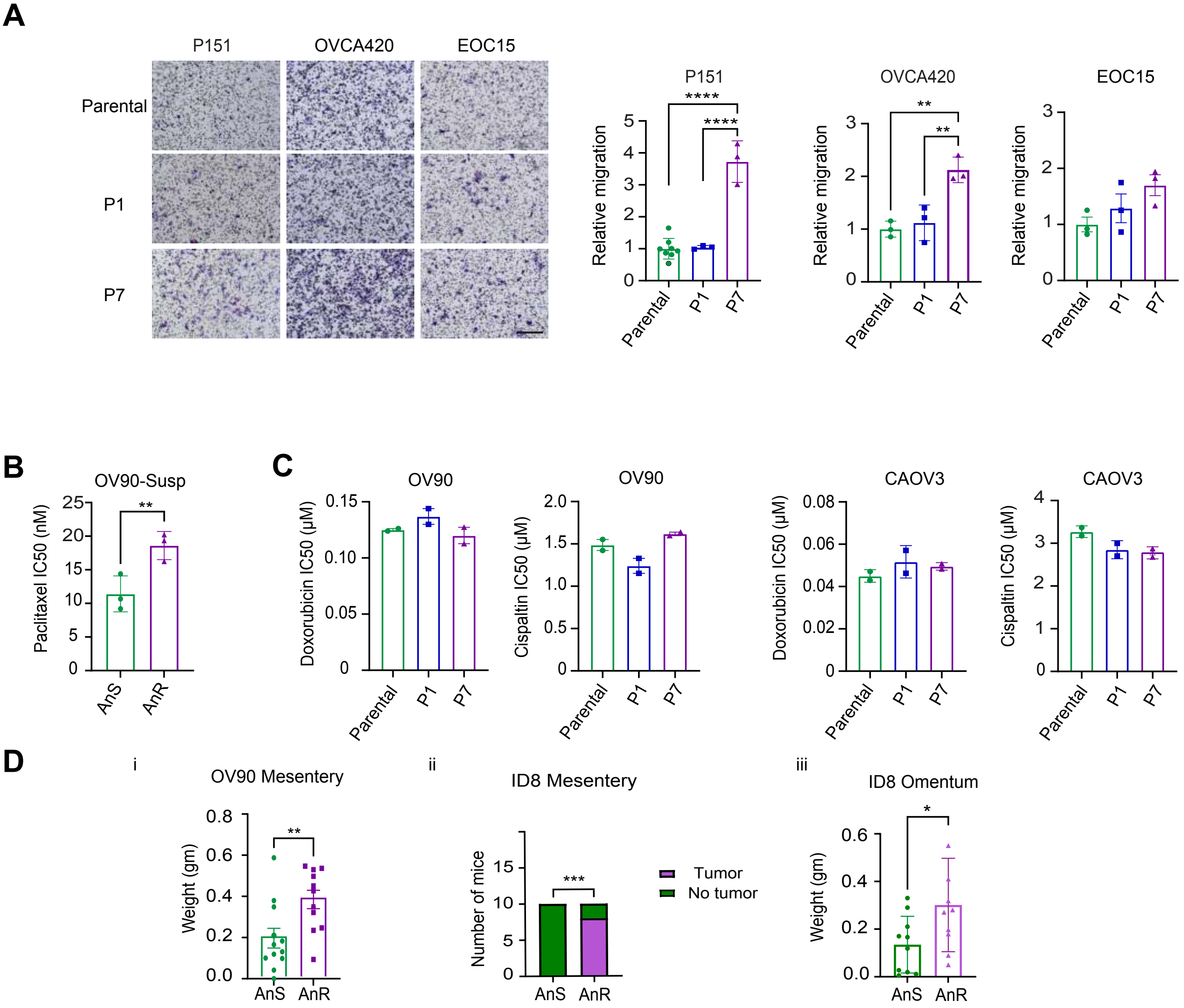
A
P151
OVCA420
EOC15
Parental
P1
P7
P151
****
****
Relative migration
0
1
2
3
4
5
Parental
P1
P7
OVCA420
**
**
Relative migration
0
1
2
3
EOC15
Relative migration
0
1
2
3
B
OV90-Susp
**
Paclitaxel IC50 (nM)
0
5
10
15
20
25
AnS
AnR
C
OV90
Doxorubicin IC50 (µM)
0
0.05
0.10
0.15
OV90
Cispaltin IC50 (µM)
0
0.5
1.0
1.5
2.0
CAOV3
Doxorubicin IC50 (µM)
0
0.02
0.04
0.06
0.08
CAOV3
Cispaltin IC50 (µM)
0
1
2
3
4
D
i
OV90 Mesentery
**
Weight (gm)
0.0
0.2
0.4
0.6
0.8
AnS
AnR
ii
ID8 Mesentery
***
Number of mice
0
5
10
15
Tumor
No tumor
iii
ID8 Omentum
*
Weight (gm)
0.0
0.2
0.4
0.6

Supp Figure 3:

(A). Representative images (Left) and quantitation (right graph) of indicated parental, P1(cells expanded after one 24 hr. exposure to suspension culture) and P7 (AnR) cells on fibronectin coated transwell filters after 24 hrs. of migration (n=3). Data are Mean ± SEM, ns $p > 0.05$,** $p < 0.01$, ****$P < 0.0001$. One way ANOVA followed by Tukey's multiple comparison. Scale bar: 200μm. (B). IC50 of indicated OV90 cell lines to paclitaxel determined under suspension culture conditions over a period of 72 hrs. using CellTiter-Glo 3D cell viability assay. Data are Mean ± SEM, ** $p < 0.01$, unpaired t test. (C) IC50 of indicated parental, P1 (cells expanded after one 24 hr exposure to suspension culture) and P7 (AnR) cells to doxorubicin and cisplatin under steady attached conditions over a period of 72 hrs. using an SRB assay. Data are Mean ± SEM, ns $p > 0.05$. One way ANOVA followed by Tukey's multiple comparison. (D). (i) mesenteric weight (ii) no. of mice with tumors in the mesentery (iii) weight of omentum in indicated cell lines from receiving either parental/AnS (P0) or AnR (P7) isogenic derivatives as indicated. OV90, n=12 for AnS and n=11 for AnR; ID8, n=10. All data are Mean ± SEM; * $p < 0.05$,** $p < 0.01$, ***$p < 0.001$, unpaired t test.

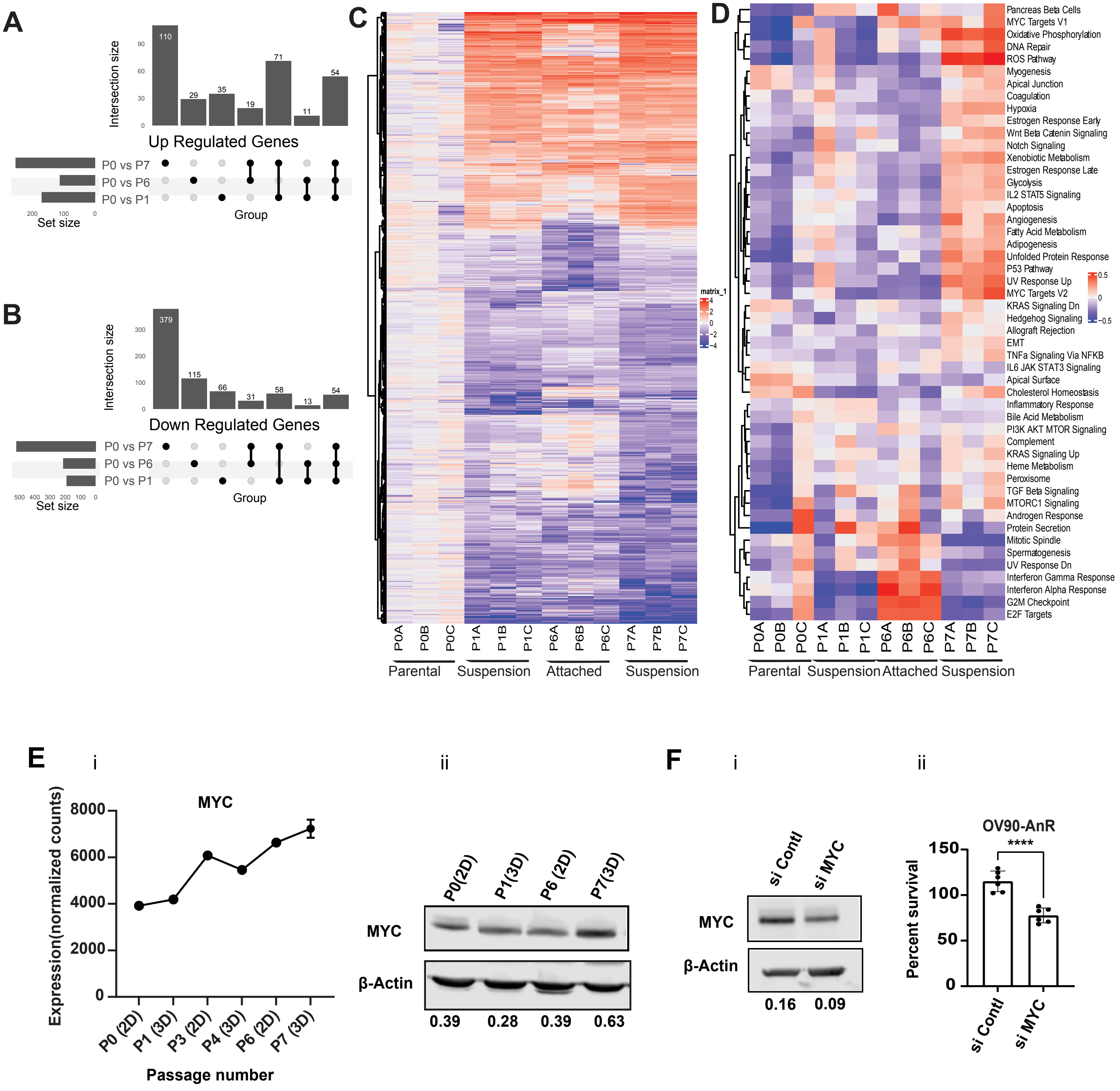
A
Intersection size
110
29
35
19
71
11
54
Up Regulated Genes
P0 vs P7
P0 vs P6
P0 vs P1
Set size
Group
B
Intersection size
379
115
66
31
58
13
54
Down Regulated Genes
P0 vs P7
P0 vs P6
P0 vs P1
Set size
Group
C
matrix_1
P0A P0B P0C P1A P1B P1C P6A P6B P6C P7A P7B P7C
Parental
Suspension
Attached
Suspension
D
Pancreas Beta Cells
MYC Targets V1
Oxidative Phosphorylation
DNA Repair
ROS Pathway
Myogenesis
Apical Junction
Coagulation
Hypoxia
Estrogen Response Early
Wnt Beta Catenin Signaling
Notch Signaling
Xenobiotic Metabolism
Estrogen Response Late
Glycolysis
IL2 STAT5 Signaling
Apoptosis
Angiogenesis
Fatty Acid Metabolism
Adipogenesis
Unfolded Protein Response
P53 Pathway
UV Response Up
MYC Targets V2
KRAS Signaling Dn
Hedgehog Signaling
Allograft Rejection
EMT
TNFa Signaling Via NFKB
IL6 JAK STAT3 Signaling
Apical Surface
Cholesterol Homeostasis
Inflammatory Response
Bile Acid Metabolism
PI3K AKT MTOR Signaling
Complement
KRAS Signaling Up
Heme Metabolism
Peroxisome
TGF Beta Signaling
MTORC1 Signaling
Androgen Response
Protein Secretion
Mitotic Spindle
Spermatogenesis
UV Response Dn
Interferon Gamma Response
Interferon Alpha Response
G2M Checkpoint
E2F Targets
P0A P0B P0C P1A P1B P1C P6A P6B P6C P7A P7B P7C
Parental
Suspension
Attached
Suspension
E
i
MYC
Expression(normalized counts)
8000
6000
4000
2000
0
P0 (2D)
P1 (3D)
P3 (2D)
P4 (3D)
P6 (2D)
P7 (3D)
Passage number
ii
P0(2D)
P1(3D)
P6 (2D)
P7(3D)
MYC
β-Actin
0.39
0.28
0.39
0.63
F
i
si Contl
si MYC
MYC
β-Actin
0.16
0.09
ii
OV90-AnR
Percent survival
150
100
50
0
****
si Contl
si MYC

Supp Figure 4:

(A). UpSet plots indicating the number of up regulated genes (p value < 0.05 and L2FC >1.5) and (B) down regulated genes (p value < 0.05 and L2FC < -1.5) in CAOV3 across the time point comparisons P0 vs P1, P0 vs P6, and P0 vs P7. (C) Heatmap for the individual CAOV3 samples using Log2FC as calculated for individual biological replicates for parental P0, (P0A-C), P1 (P1A-C), P6 (P6A-C), and P7 (P7A-C). Respective attached and suspension time points are indicated. Heatmap generated by clustering analysis of DEGs across samples (genes= 1041). The Log2 fold changes of each gene were clustered based on euclidean distance. (D). Heatmaps for the individual CAOV3 samples using GSVA normalized enrichment scores for passage 0 (P0A-C), 1 (P1A-C), 6 (P6A-C), and 7 (P7A-C). The hallmarks were clustered based on euclidean distance. (E) (i) c-MYC expression (normalized count from RNA sequencing) in indicated OV90 cells cultured using model of cyclic gain and loss of attachment. (ii) Representative western blot for c-MYC in indicated OV90 cells. Quantitation of c-MYC normalized to β-actin shown below. (n= 2). (F). (i) Representative western blot for c-MYC in P7/AnR OV90 cells following transient knockdown using siRNA against c-MYC. Quantitation of c-MYC normalized to β-actin shown below. (ii) Percent live cell count of OV90 from P7 after 24 hours in suspension assessed by Trypan blue exclusion assay following transient knock down of c-MYC. Data are Mean ± SEM; ****p < 0.0001., unpaired t test.

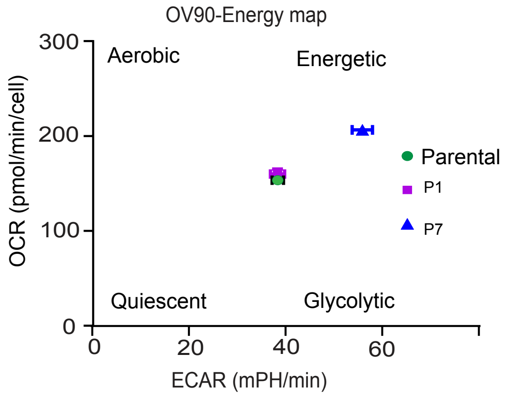
OV90-Energy map
Aerobic
Energetic
Quiescent
Glycolytic
Parental
P1
P7
OCR (pmol/min/cell)
ECAR (mPH/min)
0
100
200
300
0
20
40
60

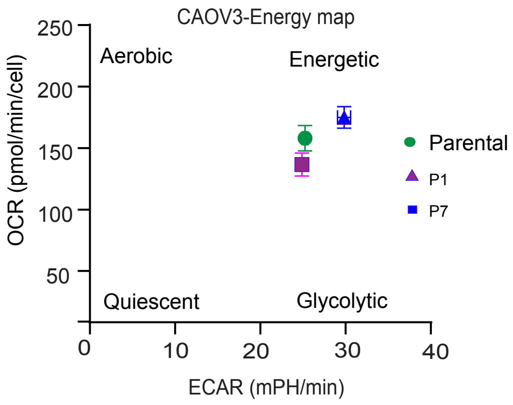
CAOV3-Energy map
Aerobic
Energetic
Quiescent
Glycolytic
Parental
P1
P7
OCR (pmol/min/cell)
ECAR (mPH/min)
50
100
150
200
250
0
10
20
30
40

Supp Figure 5:

Energy map of indicated OV90 and CAOV3 cells under attached conditions measured using the mito. stress assay on seahorse XFe96 extracellular flux analyzer.

A

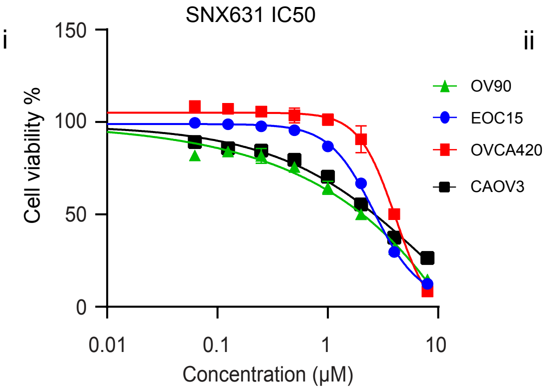

ii

Cell line	IC50 (µM)
OV90	2.16
CAOV3	2.9
OVCA420	4.11
EOC15	2.62

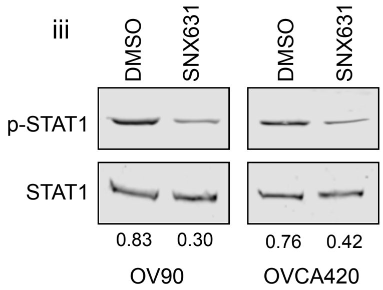

B

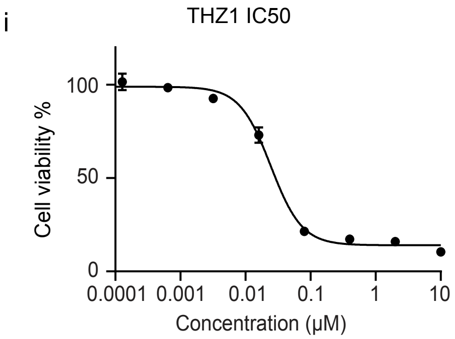

Cell line	THZ1 Mean IC50 (µM)
OV90	0.034

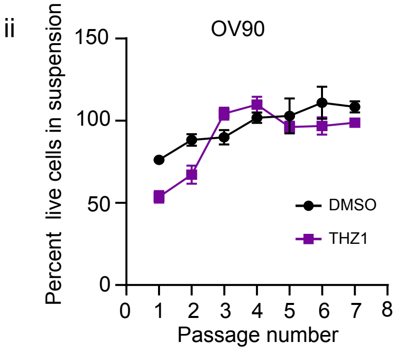

Supp. Figure 6:

(A) (i) IC50 of indicated cell lines assessed after 7 days of incubation with either vehicle or SNX631 using an SRB assay (ii) Calculation of IC50 from (i) (iii) pSTAT1 in OV90 and OVCA420 cells in response to 500nM SNX631 treatment for 24 hrs. (B) (i) IC50 of OV90 cells assessed after 3 days of incubation with either vehicle or THZ1 using an SRB assay. Adjacent table with calculation of IC50 from (i). (ii) Percent survival of OV90 cells measured by trypan blue staining after 24 hours in suspension following cycles of gain and loss of attachment as in Fig 1B, either with vehicle (DMSO) or in the presence of 17nM THZ1 (n= 3).
